# Personal biosecurity among livestock producers and veterinarians in Armenia and the Republic of Moldova

**DOI:** 10.3389/fvets.2026.1784276

**Published:** 2026-05-08

**Authors:** Mafalda Pedro Mil-Homens, Daniel Beltran-Alcrudo, Alberto Allepuz

**Affiliations:** 1 Animal Health and Anatomy Department, Faculty of Veterinary Medicine, Universitat Autònoma de Barcelona, Barcelona, Spain; 2 Food and Agriculture Organization of the United Nations (FAO), Regional Office for Europe and Central Asia, Budapest, Hungary

**Keywords:** Armenia, biosecurity, Republic of Moldova, ruminant farms, zoonoses

## Abstract

Livestock producers and veterinarians are at high risk of zoonotic disease exposure, yet protective practices are often inconsistent. Cross-sectional surveys were conducted in Armenia (387 livestock producers and 113 veterinarians) and the Republic of Moldova (373 livestock producers and 100 veterinarians). To analyze how education, knowledge, and risk perceptions influence personal biosecurity practices, specifically the use of personal protective equipment (PPE), descriptive statistics and multiple correspondence analysis (MCA) were employed. The findings revealed gaps in awareness and the implementation of preventive measures, including not using PPE to handle abortion materials or carcass disposal. In addition, risky behaviors such as feeding viscera to pets and selling unpasteurized milk were common. Although 40% of livestock producers reported having no formal training on zoonotic diseases, more than 80% indicated that they would like to learn more about the subject. Veterinarians' results reflected compliance with hygiene standards and a commitment to using PPE and implementing biosecurity measures. Additionally, greater self-reported knowledge on zoonotic diseases was associated with improved hygiene and more appropriate use of PPE in specific situations. The study underscores the critical role of education and effective communication between livestock producers and veterinarians in promoting safe practices. Addressing these gaps requires context-specific training and awareness, provision of affordable PPE, and institutional strengthening of veterinary services, framed within a One Health approach.

## 1 Introduction

Zoonoses are infections transmitted between animals and humans and account for over 60% of emerging infectious diseases (1–3). They remain a major global public health threat, especially in the context of livestock farming and veterinary practice, where frequent and close contact with potentially infected animals and their environments is routine (4–6). Responding to these risks requires more than isolated medical or veterinary interventions. The One Health approach, endorsed by the Food and Agriculture Organization (FAO), the World Health Organization (WHO), the World Organization for Animal Health (WOAH), and the United Nations Environment Programme (UNEP), promotes collaborative, cross-sectoral strategies that address the interdependence of human, animal, and environmental health (7, 8).

Diseases such as brucellosis (5), tuberculosis (9), anthrax (10), and echinococcosis (11) are zoonoses that pose significant public health risks to rural communities and animal health workers worldwide (12–15), causing occupational exposure, productivity losses, and public health burdens.

Preventing the transmission of zoonoses in such settings depends heavily on effective biosecurity measures, particularly at the individual level. Personal biosecurity, comprising individual-level protective behaviors such as hand hygiene, appropriate use of personal protective equipment (PPE), and safe handling of sick or dead animals, is especially crucial for frontline workers (16, 17). While guidelines exist, real-world implementation remains inconsistent, especially in resource-limited rural environments like smallholder farming systems. There is room for improvement in knowledge about biosecurity, PPE use, and zoonosis prevention (18, 19). Facilitating communication between farmers and veterinarians is essential to guarantee that information about biosecurity and disease prevention is understood and applied correctly. However, studies suggest that communication between livestock producers, and veterinarians is not always effective, facilitating the introduction and spread of diseases due to a lack of awareness or deficient implementation of biosecurity measures (18, 21). This persistent gap between awareness and practice underscores the urgent need to reinforce biosecurity education, improve access to PPE, and integrate occupational health principles into veterinary and agricultural policy frameworks (18, 22, 23). As zoonoses continue to pose serious threats to human and animal health, the promotion and adoption of personal biosecurity measures must be prioritized as a cost-effective, evidence-based strategy to protect those most at risk (23).

In 2024, a baseline study conducted in the country of Georgia explored these dynamics using a standardized survey tool developed by FAO and academic partners (24). The results revealed that while many livestock producers and veterinarians self-reported good knowledge of zoonoses, this did not always translate into high compliance with recommended practices, particularly regarding the use of protective face masks, protective glasses, and the proper handling of aborted materials. The study also identified demographic factors (e.g., gender and education level) that influenced the adoption of personal biosecurity measures.

Building on this methodological foundation, the present study assessed personal biosecurity practices and perceptions among livestock producers and veterinarians in Armenia and the Republic of Moldova (hereafter “Moldova”). Specifically, the study aimed to (i) describe the use of PPE and other biosecurity practices among livestock producers and veterinarians, (ii) identify perceived barriers and motivators influencing the adoption of these practices, and (iii) explore differences between professional groups and national contexts.

The survey was adapted from the Georgian study to ensure methodological consistency across contexts. This approach enables the generation of comparable evidence across countries with similar livestock production systems, endemic zoonotic disease risks, and limited veterinary public health resources. Such evidence can support the development of targeted interventions and inform future One Health policy and programming in the region.

## 2 Materials and methods

### 2.1 Study design and setting

A cross-sectional study was conducted in Armenia and Moldova between May and July 2025. The study followed the same core methodology used in a previous assessment conducted in the country of Georgia in 2024 (24), allowing for regional comparability across Eastern Europe and the Caucasus.

### 2.2 Survey development

Structured questionnaires were collaboratively developed by experts from FAO, the Universitat Autònoma de Barcelona, and national stakeholders. Adapted from the Georgian baseline study (24), the tool was designed to collect both quantitative and qualitative information from two target populations: livestock producers, and veterinarians. The surveys, with separate but comparable versions for livestock producers and veterinarians, covered socio-demographic and professional characteristics, zoonotic disease awareness and training, farm hygiene and disinfection practices, use of PPE and risk perception, and motivators and barriers to PPE use. For the questions related to perceived barriers and motivators, they were exploratory in nature and included to capture participants' perspectives on factors influencing the adoption of personal biosecurity practices. The surveys were translated into Armenian, Romanian, and Russian, and were pre-tested with a small sample of respondents in each country (10 in Moldova and a similar number in Armenia). Feedback from pre-testing was used to improve clarity, sequencing, and cultural appropriateness. Final versions of the questionnaires are available in the Supplementary file “Data sheet 1.”

### 2.3 Sampling and recruitment of livestock producers and veterinarians

Sampling in both Armenia and Moldova followed a purposive, non-probability approach, designed to capture diversity in ruminant livestock production systems, geographic representation, and veterinary service types. Target sample sizes were informed by the sample size calculations applied in the previous Georgian study (24), which used Cochran's formula assuming a 95% confidence level, an expected prevalence of 50% for the implementation of personal biosecurity measures, and a precision of 5% for livestock producers and 10% for veterinarians.

In Armenia, the target sample included 400 livestock producers and 120 veterinarians. Ultimately, 387 livestock producers and 113 veterinarians were surveyed across six regions (marzes): Syunik, Shirak, Gegharkunik, Lori, Armavir, and Aragatsotn. Livestock producers were selected based on their active engagement in ruminant livestock production and geographic distribution across regions with high population density of cattle and small ruminants. Veterinarians were recruited from both public and private sectors, prioritizing those engaged in routine fieldwork. Interviews were conducted by two teams, comprised of one supervisor and several trained enumerators from the Strategic Development Agency (SDA), a non-governmental organization in Armenia established in 2002 that specializes in agriculture community-based projects (25).

In Moldova, the intended sample included 400 livestock producers and 100 veterinarians, with 373 livestock producers, and 100 veterinarians ultimately interviewed from all regions of the country: North, Center, South, and the Autonomous Territorial Unit of Gagauzia, covering 30 out of 32 districts (rayons). Surveys were implemented by the National Rural Development and LEADER Network (NRDLN), an organization established in 2019 dedicated to the development of rural areas (26). Nine trained enumerators were deployed across the country to ensure adequate geographic and production system coverage.

### 2.4 Data collection

Face-to-face interviews were conducted by enumerators, who received dedicated training on how to properly apply the questionnaire, using digital tablets and the KoboCollect mobile data platform (27). Interviews were conducted according to a standardized protocol, and consent forms were obtained before administering the questionnaire. Two-page leaflets on zoonoses and personal biosecurity tailored to livestock producers and veterinarians were handed out to all respondents upon completion of the survey (28, 29).

For secure data input and storage, all collected data were securely stored on the Amazon Web Services (AWS) infrastructure.

### 2.5 Data analysis

Data was exported from the KoboToolbox platform (30) and then manipulated and organized in Excel^®^. All statistical analyses were performed using RStudio^®^ version 4.5.0. (2025-04-11). Descriptive statistics were calculated separately for each country and target group. Prior to analysis, the datasets were reviewed for completeness and consistency. Missing responses were retained as missing values and excluded from analyses where appropriate, with percentages calculated based on the number of valid responses for each variable. For questions allowing multiple responses (e.g., type of production system, learning formats, perceived barriers and motivators, types of personal protective equipment used, and diseases reported), each response option was treated as a separate binary variable. Frequencies were then calculated for each response category to reflect the number of times a particular option was selected by respondents.

Socio-demographic characteristics between countries were compared using Chi-square or Fisher's exact tests as appropriate.

Multiple correspondence analysis (MCA) was performed separately for livestock producers and veterinarians to examine associations between personal biosecurity practices and demographic or contextual variables. MCA was selected because the survey data consisted primarily of categorical variables and the objective was to identify underlying structures and relationships among these variables in an exploratory manner. For both groups, variables with low variability (i.e., ≥90% of responses in a single category) or a small number of observations in a category were excluded to improve discriminatory power. The final datasets were partitioned into two thematic domains: general biosecurity practices and PPE-related behaviors. Active variables for the MCA were selected based on these filters, while socio-demographic characteristics (country, gender, age, marital status, education), type of production system (livestock producers), professional role and experience (veterinarians), working days per week (veterinarians), and knowledge of zoonoses, and knowledge of zoonoses were included as supplementary variables to allow interpretation of the factorial dimensions without influencing their construction.

The MCA was conducted using the FactoMineR package in R (31). Squared correlation ratios (η^2^) were calculated to assess how strongly variables were associated with the first two dimensions. MCA was used as an exploratory ordination method, and supplementary variables were projected to aid interpretation of the factorial space. Groups defined by selected contextual variables were visualized to facilitate interpretation of patterns across respondents. Group centroids were then calculated to summarize the position of each group in the MCA space. The results from the MCA can be observed in the Supplementary file “Data Sheet 2” and the MCA code can be consulted in the Supplementary file “Data Sheet 3.”

## 3 Results

### 3.1 Descriptive analysis

#### 3.1.1 Socio-demographic and professional characteristics

A total of 387 livestock producers and 113 veterinarians were surveyed in Armenia, and 373 livestock producers and 100 veterinarians in Moldova. Table 1 summarizes the demographics of the surveyed livestock producers and veterinarians.

**Table 1:** Description of study population of livestock producers and veterinarians.

Features	Categories	Livestock producers (%)		Veterinarians (%)	
		Armenia (*n* = 387)	Moldova (*n* = 373)	Armenia (*n* = 113)	Moldova (*n* = 100)
Age	≤ 30	2.6	6.2	10.6	6.0
	31–40	14.0	24.9	12.4	15.0
	41–50	28.4	33.2	12.4	13.0
	≥51	55.0	35.7	64.6	66.0
Gender	Female	18.1	22.5	6.2	20.0
	Male	81.9	77.5	93.8	80.0
Marital status	Married	89.9	90.1	86.7	87.0
	Not married	7.5	6.7	13.3	2.0
	Divorced	0.3	2.7	-	5.0
	Do not want to answer	-	0.5	-	3.0
	Widowed	0.3	-	-	3.0
Level of education	Illiterate	0.3	0.5		
	Primary school	2.1	7.5		
	Middle school	3.4	48.0		
	Highschool	0.0	20.6		
	University	33.1	21.7		
	Postgraduate degree	3.1	1.6		
Role on the farm	Farm owner	99.5	91.2		
	Salaried employee	0.3	6.7		
	Other	0.3	2.1		
Time working as a livestock producers	≤ 5 years	0.5	15.3		
	6–10 years	5.4	31.1		
	11–20 years	21.7	30.8		
	≥21 years	72.4	22.8		
Number of workers	0	6.7	4.9		
	1–5	90.4	88.5		
	6–10	2.8	4.6		
	≥11	-	2.1		
Visits to ruminant farms	Daily			69.9	21.0
	1–2 times per week			8.0	56.0
	3–4 times per week			22.1	23.0

The majority of livestock producers in Armenia (82%) and Moldova (78%) were male. More livestock producers in Armenia were over 50 years old (55%) than in Moldova (36%) and had more experience, with 72% reporting over 21 years in the field, vs. 31% in Moldova reporting 6–10 years. In terms of education, 33% of Armenian livestock producers had university degrees, while in Moldova, 22% of livestock producers attended university.

The majority of the surveyed veterinarians were male (94% in Armenia, 80% in Moldova) and over 50 years old (65% and 66%, respectively). Daily farm visits were more common in Armenia (70%), whereas 56% of Moldovan veterinarians visited farms 1–2 times per week (Table 1).

##### 3.1.1.1 Farm and work characteristics

In Armenia, cattle production for mixed purposes (35%) and for dairy (30%) was common, while Moldova had more dairy (30%) and sheep farms (28%). Cleaning and disinfection of the farm were performed daily by 75% of Armenian livestock producers compared to 39% in Moldova. Equipment was cleaned after each use by 41% of livestock producers in Armenia and 75% in Moldova.

##### 3.1.1.2 Zoonotic awareness and experience

In Armenia, 95% of livestock producers believed zoonotic transmission was possible, compared to only 42% in Moldova. Brucellosis (94%), anthrax (70%), and dermatophytosis (56%) were the most cited diseases in Armenia. In Moldova, anthrax (29%), brucellosis (23%), and tuberculosis (20%) were most frequently mentioned. Thirty-three Armenian livestock producers reported contracting brucellosis, and four of those also had dermatophytosis. Veterinarians in Armenia, rated their zoonosis knowledge as 9 out of 10 (34%) and in Moldova 40% rated it as 8. These professionals reported contracting brucellosis, cowpox, dermatophytosis (Armenia), and dermatophytosis and rabies exposure (Moldova).

##### 3.1.1.3 Biosecurity practices

In Armenia, 57% of livestock producers reported having access to changing rooms, 60% had running water, and 64% used liquid soap for handwashing. Alcohol-based disinfectant usage stood at 56%, though only 2% utilized disposable towels. A total of 69% of livestock producers stated that they washed their farm clothing daily. In Moldova, 64% had access to changing rooms, 81% had running water, and 97% used liquid soap. Alcohol disinfectant use was at 54%, while 24% of Moldovan livestock producers used disposable towels, and only 47% reported washing their farm clothing daily.

Regarding hand hygiene, virtually all livestock producers (100% in Armenia and 99% in Moldova) always washed their hands after coming into contact with animal body fluids. Additionally, 100% of Armenian livestock producers and 92% of Moldovan livestock producers ensured they washed their hands before eating, drinking, or smoking. Among veterinarians, 100% of respondents in Armenia always washed their hands before eating, drinking, or smoking; meanwhile, in Moldova, all veterinarians stated they always washed their hands after handling animal body fluids. Responses regarding food safety found that 75% of livestock producers in Armenia and 63% in Moldova regularly sold unpasteurized milk or cheese. More than 50% of livestock producers in both countries indicated that they never fed their pets viscera or allowed them to roam the farm. When dealing with sick animals, over 90% of livestock producers in both Armenia and Moldova reported they either contacted a veterinarian or personally treated the animals (Table 2).

**Table 2:** Practices of livestock producers and veterinarians related to zoonoses and livestock health.

Hand washing	Livestock producers (%)						Veterinarians (%)					
	Armenia (*n* = 387)			Moldova (*n* = 373)			Armenia (*n* = 113)			Moldova (*n* = 100)		
	Always	Sometimes	Never	Always	Sometimes	Never	Always	Sometimes	Never	Always	Sometimes	Never
After contact with animal body fluids	100			99	1	0	99	1	0	100	0	0
Before contact with animals	80	16	4	86	13	1	88	8	4	94	5	1
Before eating/drinking/smoking	100			92	8	0	100	0	0	99	1	0
If gloves are used, wash hands after contact with animals	98	2	0	65	17	18	95	4	1	92	7	1
Use waterproof bandages	97	2	1	70	29	1	94	5	1	96	4	0
Wash the wound site after getting a cut	98	1	0	81	18	0	100	0	0	98	2	0
After removing PPE							99	1	0	93	7	0
After contact with equipment contaminated with animal body fluids							99	1	0	99	1	0
Consumption												
Boil/pasteurize milk before consumption	97	2	1	45	42	13						
Clean/safe water supply for consumption	98	1	1	93	6	1						
Consume raw/undercooked meat	4	5	91	1	2	97						
Dispose of animal waste/dead animals in nearby fields	28	5	66	2	2	96						
Sell unpasteurized milk/cheese	75	13	12	63	17	19						
Wash fruits/vegetables before eating/cooking	99	1	0	82	17	1						
Pets												
Allow pets on the farm	12	21	67	11	39	50						
Feed pets viscera	8	27	65	14	35	50						
Regularly apply antiparasitic medicines to pets	99	1	0	83	15	3						
Sick animals												
Call the veterinarian	99	1	0	92	7	1						
Isolate sick animals	59	35	6	84	14	2						
Treat infected animals	99	1	0	96	3	1						
Vaccinate animals against zoonosis	100	0	0	97	2	1						

##### 3.1.1.4 Personal protective equipment (PPE) use

When handling sick or suspected animals, 95% of Armenian livestock producers used overalls and boots, but only 56% used gloves. In Moldova, these rates were lower at 36% and 26%, respectively. Among veterinarians, the use of overalls and gloves for most situations while handling animals or animal body fluids was common, with 95% in Armenia and 87% in Moldova (Table 3).

**Table 3:** Livestock producer and veterinarian personal protective equipment implementation.

Contact with animals or animals fluids	Livestock producers (%)												Veterinarians (%)									
	Armenia (*n* = 387)						Moldova (*n* = 373)						Armenia (*n* = 113)					Moldova (*n* = 100)				
	O	B	GLO	FM	PG	NP	O	B	GLO	FM	PG	NP	O	B	GLO	FM	PG	O	B	GLO	FM	PG
Healthy animals	94	94	41	1	0	1	34	36	20	3	1	3	99		96	14	0	88	90	34	10	3
Sick animals	95	94	56	6	0	0	36	38	27	4	1	1	100		98	30	2	93	99	70	33	0
Animals suspected of having a disease	95	95	57	10	0	0	35	37	25	3	1	2	100		100	39	10	99	99	79	45	0
Disposal of carcasses	95	95	74	9	0	0	37	40	30	7	1	1	100		100	61	6	99	100	78	44	2
Parturition	95	94	80	5	0	0	35	39	25	2	1	1	100		96	35	2	93	94	60	28	2
Disposal of abortion stillbirths	95	95	88	5	0	0	34	39	29	3	0	1	99		100	50	5	95	99	69	38	0
Cleaning surfaces/stables	95	95	86	55	2	0	37	42	24	4	0	1										
Postmortem exam													100		99	39	7	98	99	83	49	0
Blood, feces, or fluids of animals													100		100	36	4	96	100	69	36	0
Surgery													100		99	58	4	94	100	79	45	0
Vaccination													100		98	26	3	90	98	48	24	2
Blood sampling													99		98	26	2	91	98	54	26	1
Treatment													100		96	21	0	91	99	50	23	1

^*^B, Boots; FM, Face mask; PG, Protective glasses; GLO, Gloves; NP, No Personal protective equipment; O, Overalls.

##### 3.1.1.5 Perception of zoonotic risk

In Armenia, 80% of livestock producers believed it was likely to contract diseases through contact with sick animals, aborted materials, and dead animals, while only 5% identified contact with healthy animals as a risk. In Moldova, 80% believed disease transmission through cleaning stables or surfaces, or by contact with healthy animals was unlikely. Around 55% of livestock producers perceived contact with sick or suspected animals as a risk (Table 4).

**Table 4:** Livestock producers' perception of zoonotic risk associated with specific farm tasks.

Perception of zoonotic risk	Armenia (*n* = 387) (%)			Moldova (*n* = 373) (%)		
	Very likely	Likely	Unlikely	Very likely	Likely	Unlikely
Cleaning surfaces/stables	1	73	26	2	17	82
Contact with an animal suspected of having a disease	14	85	2	13	54	34
Contact with dead animals	13	85	2	9	44	46
Contact with healthy animals	0	5	93	1	10	89
Contact with sick animals	2	93	5	13	55	32
Disposal of abortion materials/stillbirths	12	87	1	4	40	55
Assisting parturition	10	86	4	3	39	58

##### 3.1.1.6 Motivators and barriers to personal protective equipment (PPE) use

In Armenia, 81% of livestock producers used PPE to protect their own health and reducing the risk of transmitting infections to others, with less than 10% citing discomfort or heat as barriers to its implementation. In Moldova, PPE use was motivated by self-protection (63%) and protection of others (58%), while discomfort (42%) and heat or humidity (39%) were common barriers. Among veterinarians, 72% (Armenia) and 82% (Moldova) cited personal protection and protection of others as the motivations for PPE use. Most (94%) disagreed with the idea that PPE use was unnecessary, not important, or poorly perceived by livestock producers (Table 5).

**Table 5:** Motivators and barriers for using personal protective equipment.

Motivators to use PPE	Livestock producers (%)						Veterinarians (%)					
	Armenia (*n* = 387)			Moldova (*n* = 373)			Armenia (*n* = 113)			Moldova (*n* = 100)		
	SA	A	D	SA	A	D	SA	A	D	SA	A	D
Colleagues convinced	47	11	41	4	18	78	14	9	77	18	24	58
Farmers expectation							67	28	4	60	29	11
The individual already has health problems	50	5	44	6	17	77	17	12	71	25	27	48
It is the individual's responsibility							73	26	2	74	25	1
Improve animal productivity	78	20	2	32	24	44	69	29	2	57	27	16
Improve animal health/welfare	80	20	1	38	28	34	70	28	2	70	28	2
Previous zoonotic disease experience							16	5	79	24	13	63
Recommended by regulations	67	20	13	23	41	36	55	39	6	43	52	5
Prevent disease introduction	80	19	1	38	35	27	67	26	7	72	28	0
Protect the individual's health	81	19	0	63	36	1	72	28	0	85	15	0
Protect others from becoming infected	81	18	1	58	38	4	73	27	1	83	17	0
Feels protected from disease							73	25	2	72	27	1
Keep protective clothing clean							27	13	59	61	32	7
Vet advised	49	17	34	24	38	38						
Animals the farmer works with are infected with zoonosis	78	10	13	5	12	83						
Know others who got infected	53	14	34	2	9	89						
Barriers to PPE use												
Do not know how and when to use it	0	0	99	3	6	92	0	2	10	0	3	97
Difficult to use	6	8	86	4	19	77	3	16	81	3	11	86
Expensive	0	3	97	5	27	68	0	3	97	2	20	78
Time consuming	7	7	86	7	28	65	1	17	82	2	11	87
Too hot or too humid	9	9	82	14	39	47	4	16	81	4	37	59
Uncomfortable	9	16	75	11	42	46	4	16	81	4	35	61
Not important	2	9	90				0	5	95	0	4	96
Never thought of using it	1	7	92				0	4	96	0	1	99
Requirements of farmers							3	19	78			
Negative perception of farmers							0	1	99	5	26	69
PPE does not fit							0	14	86	13	24	63
PPE provided by the farm is visibly dirty							0	14	86	10	25	65
Colleagues think I am too cautious							1	21	91	2	10	88

^*^SA, Strongly agree; A, Agree; D, Disagree.

##### 3.1.1.7 Education and training

In Armenia, 73% of livestock producers had received zoonosis training, and 85% expressed interest in learning more, with 66% preferring workshops for training. In Moldova, 47% had received training, 80% wanted to learn more, of which 76% preferred face-to-face training. After obtaining their initial veterinary qualifications, 80% of Armenian and 67% of Moldovan respondents reported receiving training on zoonoses. In both countries, over 93% expressed interest in further education, with a preference for workshop-based and face-to-face formats (Table 6).

**Table 6:** Education and training on zoonosis and zoonosis prevention for livestock producers and veterinarians.

Training	Livestock producers (%)		Veterinarians (%)	
	Armenia (*n* = 387)	Moldova (*n* = 373)	Armenia (*n* = 113)	Moldova (*n* = 100)
Received training on zoonosis and zoonosis prevention	73	47	80	67
Interested in learning about zoonosis and zoonosis prevention	84	79	93	93
Format of training received				
Face-to-face	51	76	59	84
Workshop	66	16	89	41
Online	3	29	40	32
Leaflets or brochures	49	16	28	27
Short videos	34	27	17	28
TV	9	7	13	14

### 3.2 Univariate comparisons of socio-demographic characteristics between countries

Significant differences between countries are observed across several variables (all *p* < 0.001), including perception and knowledge of zoonoses (Armenia: 95% “Yes” vs. Moldova: 42%), education (assessed using Fisher's exact test due to small cell counts), production type (Armenia primarily “cattle only” or mixed systems, whereas Moldova is largely “small ruminants only”), age distribution (with Armenia skewing older), marital status (Fisher's exact test), and livestock experience (Armenia predominantly ≥21 years vs. Moldova more often < 10 years). The number of workers also differs significantly (Fisher's exact test, *p* = 0.003), with Moldova including a small subgroup of farms with ≥10 workers (2.1%), while Armenia has none (Supplementary file “Data sheet 2”).

Concerning veterinarians, gender distribution differs significantly between countries (χ^2^ = 9.134, df = 1, *p* = 0.0025), with a higher proportion of female veterinarians in Moldova (20%) than in Armenia (6.2%). Armenian veterinarians are significantly more likely to report working with beef cattle (100% vs. 71%; χ^2^ = 37.935, *p* < 0.001) and dairy cattle (99% vs. 87%; χ^2^ = 12.680, *p* < 0.001), whereas involvement with sheep and goats is similar between countries (*p* = 0.794 and *p* = 0.348, respectively). Age and years of experience do not differ significantly (both *p* ≈ 0.65), indicating comparable career-stage profiles. However, time spent on farms differs markedly (χ^2^ = 67.165, df = 2, *p* < 0.001): Armenian veterinarians more frequently report daily farm visits (70% vs. 21%), while Moldovan veterinarians more commonly spend only 1–2 days per week on farms (56% vs. 8%). Finally, knowledge of zoonoses also differs significantly (χ^2^ = 10.784, df = 2, *p* = 0.0046), with Armenian veterinarians more often falling into the highest knowledge category (scores 9–10: 34% vs. 15%) (Supplementary file “Data sheet 2”).

### 3.3 Multiple correspondence analysis

#### 3.3.1 Livestock producers

##### 3.3.1.1 Biosecurity questions

The multivariate analysis identified two main dimensions summarizing livestock producers' biosecurity practices, with Dimension 1 explaining 16% of the total variance and Dimension 2 explaining 12%. Dimension 1 primarily reflected hygiene-related behaviors, whereas Dimension 2 was mainly associated with risk-related behaviors. Washing farm-dedicated clothing showed a strong association with the first dimension (Eta^2^ = 0.388), while handwashing after using gloves had strong effects across both dimensions (Dimension 1: 0.401 and Dimension 2: 0.378). Safe milk handling practices, such as boiling or pasteurizing, were also highly influential (Dimension 1: 0.397 and Dimension 2: 0.261), whereas selling unpasteurized products had a stronger effect in the second dimension (Dimension 2: 0.446), indicating variability in risk-related behaviors. In contrast, practices like pet management and isolation of sick animals had minimal associations, suggesting inconsistent implementation of these measures (Supplementary file “Data sheet 2”).

Biosecurity behaviors differed markedly between participants in Armenia and Moldova. Armenian livestock producers reported higher daily washing of farm-dedicated clothing (69% vs. 47% in Moldova) and stronger adherence to hand hygiene, with nearly all respondents reporting hand washing after glove use (98% vs. 65%). Milk safety practices also diverged, with 97% of Armenian livestock producers always boiling milk compared to 45% of Moldovan livestock producers, though above 60% of respondents in both countries reported selling unpasteurized products. In Moldova, 96% of livestock producers never disposed of carcasses in fields, compared to 72% in Armenia. Pet management results showed that 67% of Armenian livestock producers never allowed their pets to roam on the farm, and 65% never fed them viscera. Concerning Moldova, 50% of livestock producers never allowed their pets to roam on the farm, and the same percentage never fed them viscera. The MCA biplot suggests that cross-country differences in livestock producers' response patterns are mainly structured along two latent gradients: hygiene-related practices (Dimension 1; 16% of variance) and risk-related practices (Dimension 2; 12% of variance). The partial separation of Armenia and Moldova along the MCA space indicates that livestock producers in the two countries tend to differ in their combined hygiene and risk profiles, although the substantial overlap shows considerable within-country heterogeneity (Supplementary file “Presentation 1–Figure 1”).

Education influenced biosecurity adherence. Livestock producers with university degrees reported more consistent use of disinfectants (72%), daily washing of clothing (61%), and boiling or pasteurizing of milk (78%) than those with only primary school education. Higher education was also correlated with stricter animal management, such as limiting pet access to the farm (59%) and isolating sick animals (63%). However, risky behaviors, particularly selling unpasteurized products, remained common across all education levels, suggesting that knowledge alone does not always translate into safer practices. The MCA biplot shows that the points representing different education categories largely overlap across the MCA space, indicating that education level alone does not strongly structure livestock producers' combined biosecurity practices. Instead, there is substantial within-education heterogeneity. Any small tendencies for particular education groups to concentrate in certain areas of the plot appear weak compared with the overall overlap, suggesting that other factors likely contribute more to the observed variability in responses (Supplementary file “Presentation 1–Figure 2”).

Perceived zoonotic risk played a central role in shaping biosecurity behaviors. Livestock producers that were aware of zoonoses risks demonstrated better hygiene (57%), more frequent washing of farm-dedicated clothing (59%), and milk boiling or pasteurization (81%) than those uncertain or dismissive of the risks of zoonosis. However, even among respondents aware of zoonoses, inconsistent practices were noted in waste disposal (31% fed their pets viscera) and selling unpasteurized milk or cheese (71%). Those uncertain about zoonotic transmission risks were the least compliant overall and reported greater variability in implementing hygiene measures. The MCA biplot shows that cluster of “Yes” points (blue) in the bottom-left indicates that livestock producers reporting awareness of zoonoses exhibit more consistent biosecurity practices along the two main MCA dimensions, consistently reporting hygiene behaviors. In contrast, “No” and “I don't know” responses are more widely spread across the plot, reflecting greater variability in both hygiene and risk practices (Supplementary file “Presentation 1–Figure 3”).

##### 3.3.1.2 Personal protective equipment (PPE) questions

The multivariate analysis of farm-level PPE use identified two main dimensions, with Dimension 1 explaining 22% of the total variance and Dimension 2 explaining 12%. Dimension 1 primarily reflected motivational drivers for PPE use, whereas Dimension 2 was mainly associated with the type of PPE used and perceived risk of contracting a zoonosis. The use of overalls showed moderate associations with most contact types (Eta^2^ ≈ 0.27–0.30), while the use of gloves exhibited very strong associations with high-risk situations, especially contact with sick animals (Dimension 2: 0.597) and those suspected of having a disease (Dimension 2: 0.602). Protective face masks were less commonly used, showing a moderate association only for cleaning surfaces (Dimension: 0.276). The highest associations were linked to motivations for PPE use, including improving animal productivity (Dimension 1: 0.705), preventing disease introduction (Dimension 1: 0.662), and following regulations (Dimension 1: 0.608). Practical constraints such as cost, discomfort, or time burden had low influence (Eta^2^ < 0.25), indicating that protective behavior was mainly driven by perceived benefits rather than barriers (Supplementary file “Data sheet 2”).

Marked differences in PPE use were found between Armenia and Moldova. Armenian livestock producers reported consistently higher PPE use across all contact scenarios, with over 90% wearing overalls and over 80% using gloves during high-risk tasks, compared to 76–83% and 39–58%, respectively, in Moldova. Concerning mask use, 55% of Armenian livestock producers reported mask use for surfaces or stables' cleaning, compared with only 6% of Moldovan livestock producers. Both Armenian and Moldovan respondents expressed preventive and social motivations to use PPE, such as protecting others' health (81% Armenia; 58% Moldova). PPE practices between Armenia and Moldova. Armenian farmers (pink) cluster primarily on the left of Dimension 1, indicate more uniform adoption of PPE motivated by improving animal health and animal productivity goals. In contrast, Moldovan farmers (blue) are more dispersed and extend strongly to the right, reflecting a wider variety of PPE responses and adoption patterns. These results indicate that the main difference between countries lies in general PPE motivations and exposure-related practices (Dimension 1), while variation within each country is more evident in situation-specific PPE use (Dimension 2) (Supplementary file “Presentation 1–Figure 4”).

Among livestock producers, those with university degrees reported broad use of overalls (>95%) and higher glove adoption (39%−88%) across all tasks, whereas those with only primary school education reported lower and more inconsistent use. Higher education was also associated with livestock producers having a stronger perception of the potential contact scenarios that could be a risk for disease introduction. The MCA biplot shows that the education groups largely overlap across the space, indicating that education alone does not strongly structure PPE-related practice profiles and that there is substantial variability within each education category (Supplementary file “Presentation 1–Figure 5”).

Perception of zoonotic risk was a major behavioral determinant. Livestock producers that acknowledged zoonotic hazards reported the highest PPE use, over 90% for overalls and 80% for gloves in risky scenarios, compared to lower rates among uncertain or dismissive respondents. Both livestock producers who acknowledged zoonotic risks and those who were unaware of them stated that PPE was useful for protecting others (75% and 62%, respectively), preventing disease introduction (68% and 45%), and improving animal health or welfare (68% and 46%, respectively). The MCA biplot shows patterns in PPE responses according to zoonosis perception. Along Dimension 1 (21.7%), driven mainly by broad PPE motivations and perceived exposure, such as improving animal health and productivity, and following regulations, livestock producers answering “Yes” (blue) cluster more on the left/upper-left, while “No” (green) and especially “I do not know” (red) tend to appear toward the right. This indicates that awareness of zoonoses is linked to a more coherent PPE profile along this motivation/exposure gradient, although some overlap highlights variability within each group. Dimension 2 (12%), associated with situation-specific PPE use during high-risk tasks (e.g., handling sick, suspect, or dead animals), shows variation across all three perception categories, suggesting that targeted PPE practices differ widely regardless of overall zoonosis awareness (Supplementary file “Presentation 1–Figure 6”).

#### 3.3.2 Veterinarians

##### 3.3.2.1 Biosecurity questions

The multivariate analysis of veterinarians' biosecurity practices identified two dominant dimensions, with Dimension 1 explaining 35% of the total variance and Dimension 2 explaining 33%. Both dimensions were primarily driven by hygiene practices related to clothing. Washing frequency displayed strong associations in both dimensions (Eta^2^ = 0.518 in Dimension 1 and 1.0 in Dimension 2). The washing location also contributed (Eta^2^ = 0.518 in Dimension 1), confirming that both factors are central to veterinarians' biosecurity routines. Overall, frequent and consistent clothing hygiene emerged as a key indicator of biosecurity adherence (Supplementary file “Data sheet 2”).

Clear country differences were observed. In Armenia, almost all veterinarians (90.3%) reported daily washing of farm-dedicated clothing, compared with 60% in Moldova, where 38% washed clothing weekly and 2% did so rarely. Most Armenian respondents (98.2%) washed farm clothing at home, while in Moldova, 77% did so at home and 23% at the workplace. The MCA biplot shows that Moldovan veterinarians (blue) cluster far to the right, whereas Armenian veterinarians (pink) are closer to the center/left, highlighting distinct approaches to washing and handling farm-dedicated clothing (Supplementary file “Presentation 1–Figure 7”).

Self-reported knowledge of zoonoses was strongly associated with better hygiene practices. Among veterinarians with high self-reported knowledge scores (9–10), 86.8% washed farm-dedicated clothing daily, compared with 69.7% among those with low knowledge (1–4). The frequency of washing the farm clothing at home also increased with self-reported knowledge on zoonosis, from 84.8% among respondents that reported low awareness to 92.5% among those with high awareness. The MCA biplot shows that veterinarians with high zoonosis knowledge scores (9–10) are positioned far to the right on Dimension 1, whereas those with low knowledge scores (1–4) are closer to the center/left, indicating distinct patterns in clothing-related biosecurity practices across knowledge levels. Veterinarians with moderate knowledge (5–8) tend to fall between these two regions (Supplementary file “Presentation 1–Figure 8”).

##### 3.3.2.2 Personal protective equipment (PPE) questions

The multivariate analysis of veterinarians' use of PPE identified two main dimensions, with Dimension 1 explaining 36% of the total variance and Dimension 2 explaining 11%. Both Dimension 1 and Dimension 2 were primarily driven by the use of PPE for specific tasks. Protective glasses use showed high Eta^2^ values in Dimension 1 for animal fluids (Dimension 1: 0.577, Dimension 2: 0.122), handling dead animals (Dimension 1: 0.561, Dimension 2: 0.146), blood sampling (Dimension 1: 0.535, Dimension 2: 0.113), and postmortem contact (Dimension 1: 0.519, Dimension 2: 0.156), while mask use was most strongly linked to sick animals (Dimension 1: 0.531, Dimension 2: 0.120), vaccination (Dimension 1: 0.518, Dimension 2: 0.084), and treatments (Dimension 1: 0.501, Dimension 2: 0.134). Perceived exposure to zoonosis (Eta^2^ < 0.234) and personal motivations to use PPE (Eta^2^ < 0.20) showed weaker associations (Supplementary file “Data sheet 2”).

Mask use in Armenia was most frequently implemented to handle dead animals (61%), while in Moldova, it was used most often for postmortem activities (83%). Protective glasses, were most often used for postmortem examination (49%) in Moldova and for handling animals suspected of being sick (9.7%) in Armenia. Despite these differences, personal health was the main motivation for PPE use in both countries, with strong association in Armenia (71.7%) and Moldova (85%). The MCA biplot shows Armenian veterinarians (pink) are concentrated on the left side of Dimension 1, while Moldovan veterinarians (blue) are more widely spread and extend toward the right, highlighting clear differences in PPE practices between countries alongside within-country variability (Supplementary file “Presentation 1–Figure 9”).

Self-reported knowledge of zoonosis strongly influenced PPE behavior. Veterinarians with low knowledge scores (1–4) relied on protective face masks for dead animal handling (75.8%) and for postmortem examination (72.7%), and those with high knowledge scores (9–10) relied on protective face masks for dead animal handling (67.9%) and surgery (69.7%). The belief that healthy animals are unlikely to transmit zoonosis (low risk) was shared by both low (66.7%) and high (83.0%) knowledge groups, with both groups also agreeing that sick animals are likely to transmit diseases (57.6% and 58.5%, respectively). The likelihood of using PPE for personal protection increased with knowledge, reported by 63.8% of veterinarians with scores of 1–4 and 90.6% with scores of 9–10. The MCA biplot shows that veterinarians with moderate knowledge (5–8) are broadly dispersed across both dimensions, forming much of the central cloud, while high-knowledge (9–10) and low-knowledge (1–4) veterinarians are mixed in the same regions (Supplementary file “Presentation 1–Figure 10”).

## 4 Discussion

This study provided valuable insight into biosecurity practices (particularly related to the use of PPE) and levels of perceived knowledge regarding zoonotic diseases among livestock producersand veterinarians in Armenia and Moldova, building on earlier work in Georgia (24). Several important insights emerged that have implications for regional zoonoses control and occupational health policy, which underscore the need for integrated, context-sensitive interventions framed within a One Health approach.

Important gaps in biosecurity were identified among both professions. Among livestock producers, the sale of unpasteurized milk and feeding raw viscera to pets was common, while veterinarians often reported inadequate use of PPE during high-risk activities, such as not using protective face masks, or protective glasses when handling abortions. These practices pose serious risks for zoonotic transmission, underscoring the need for improved training, guidance, and enforcement of preventive measures. The findings also highlight that education plays a key role in shaping on-farm behaviors and that effective communication between livestock producers and veterinarians is essential for increasing awareness and promoting safer practices.

### 4.1 Livestock producers

In this study, education level played a central role in shaping attitudes and behaviors, and biosecurity and PPE behaviors were strongly stratified by education level. These results align with other studies, that associate higher education levels with a better understanding of biosecurity implementation and zoonotic diseases (19, 20). Selling or consuming unpasteurized milk presents a direct public health hazard (32), and diseases such as brucellosis, salmonellosis, and Q-fever can be transmitted through the consumption of raw unpasteurized dairy products (33, 34). Consistent with other research (34, 35), this study demonstrates that these practices persist on cattle and small ruminant farms, especially among those with lower education levels or limited understanding of zoonotic risks. These findings emphasize the need for targeted training for livestock producers to increase awareness and understanding of zoonotic diseases, transmission routes and best practices for handling dairy products to improve farm-level biosecurity (33, 36). Indeed, building awareness around zoonotic risk is essential, not only to protect individual livestock producers but also to prevent wider public health threats from emerging through unsafe on-farm practices.

In the current study, another behavior linked to livestock producers with limited formal education was the feeding of viscera to pets and allowing them to roam freely, which raises the risk of pathogen transmission within and beyond the farm environment. Echinococcosis is a disease that can have a major impact on human health, and both ruminants and domestic or wild canids play a crucial role in the parasite life cycle (37, 38). A systematic review showed that, between 1997 and 2021, there were 64,745 human cystic echinococcosis cases reported from 40 European countries, with one large-scale study estimating approximately 8,000 and 37,000 cystic echinococcosis infections in rural endemic areas in Bulgaria and Romania, respectively (11). This cycle of transmission between pets, livestock, and humans underscores the need for improved awareness and control measures, as diseases can spread through this interconnected network, posing significant risks to public health.

Perceptions of zoonotic risk also differentiated livestock producers' practices. Producers who believed animals can transmit disease to humans were more likely to adopt safer practices when handling sick or dead animals, than those who either disagreed or were uncertain that animals pose a zoonotic risk. Dealing with abortion materials and dead animal carcasses is a practice associated with a high risk of contracting diseases such as brucellosis, anthrax, or leptospirosis (10, 33, 39). The appropriate use of PPE, along with cleaning and disinfection of tools, equipment, and hands, is essential to reduce this risk (40, 41), as is carcass disposal via approved methods, such as burial in deep pits away from water sources, incineration, or rendering (41, 42).

### 4.2 Veterinarians

While veterinarians generally reported implementing hygiene practices, risky behaviors persisted.

Veterinarians' perceived knowledge of zoonoses was found to influence both biosecurity and PPE practices. Those with higher self-reported knowledge scores adhered more to consistent hygiene habits, which can reduce disease transmission. Lower perceived knowledge in veterinarians was associated with inconsistent PPE usage, frequently limited to specific situations (e.g., handling dead animals) compared to higher-knowledge counterparts, suggesting that increased understanding enables veterinarians to tailor PPE use to specific situational risks. Both low- and high-knowledge groups mentioned it was unlikely to get a disease from apparently healthy animals, which can be an issue due to subclinical infections. Moreover, the motivation to use PPE for self-protection was higher among knowledgeable veterinarians, underscoring the role of education in fostering a sense of personal responsibility and risk awareness. Overall, these findings emphasize that strengthening veterinarians' knowledge of zoonoses is essential, not only for improving hygiene and PPE compliance, but also for cultivating a more risk-aware professional culture that prioritizes both personal and public health.

### 4.3 General

The results highlight a gap between knowledge and practice with patterns of PPE use revealing both behavioral and structural barriers. For example, while most veterinarians reported high awareness of zoonoses, they also admitted rarely using masks during parturition or protective glasses during surgery. Similarly, many farmers acknowledged zoonotic risks but continued to sell raw milk or dispose of carcasses in fields, and more educated farmers reported not using alcohol-based disinfectants or failing to pasteurize milk.

Gloves and boots were commonly used, but protective face masks and protective glasses were not, despite their critical role in preventing aerosol- and droplet-transmitted infections such as brucellosis and Q fever (5). Among livestock producers, PPE use was mainly motivated by protecting personal or others' health, whereas veterinarians more often referred to professional responsibility and self-protection. This suggests that PPE adoption is not driven by knowledge alone, but also by how risks are perceived and socially framed.

PPE use is influenced by multiple factors, including comfort, usability, social norms, habit, and access to resources, which helps explain why knowledge alone does not consistently translate into practice. In this study, most livestock producers reported limited formal training on zoonoses or preventive measures. In contrast, veterinarians indicated that they provide such guidance to farmers, underscoring misalignment in communication and knowledge transfer on biosecurity practices.

Effective communication between livestock producers and veterinarians is essential to ensure that biosecurity measures are correctly implemented and that accurate, practical knowledge about zoonotic diseases is conveyed. However, when veterinarians provide inconsistent information or lack the necessary training to guide livestock producers, this creates confusion and hinders the adoption of appropriate practices on farms (18, 43). Strengthening this interface is therefore critical for improving on-farm biosecurity.

Responding to these risks requires more than isolated interventions. The One Health approach, endorsed by FAO, WHO, WOAH and UNEP, emphasizes the interconnectedness of human, animal, and environmental health. Our results illustrate why this perspective is critical: livestock producers' practices are shaped by education and community perceptions, while veterinarians' practices are influenced by institutional systems and resource constraints. In both cases, behaviors have implications beyond the individual, affecting wider public health.

Interventions should therefore combine behavior change strategies with structural support, including investment in veterinary services and infrastructure, and closer coordination between veterinary and public health sectors. Across Armenia and Moldova, the observed patterns suggest an opportunity for harmonized regional action, aligned with international One Health frameworks.

From a policy perspective, there is an urgent need to integrate personal biosecurity into national and regional zoonotic disease control strategies. Training alone is unlikely to bridge the gap between knowledge and behavior unless paired with measures that reduce the cost and increase the availability of PPE, and establish its use as a professional expectation within veterinary services and farming communities. Based on the results of the current study, several practical actions are recommended:

- Deliver targeted training and communication to livestock producers and veterinarians, emphasizing both zoonotic transmission risks and the correct use of PPE, stressing the risks of aerosol and ocular transmission. Framing protective practices as safeguarding family and community health may strengthen motivation.
- Provide or subsidize PPE, particularly protective face masks and protective glasses, through national veterinary services or livestock producers cooperatives to reduce cost barriers.
- Strengthen institutions for veterinarians by providing regular refresher training, monitoring of protective practices, and ensuring adequate PPE supplies for fieldwork.

### 4.4 Limitations

This study has several limitations that should be acknowledged. The sampling approach was purposive rather than random, which may reduce generalizability. Self-reported practices may not always align with actual behavior, which raises the possibility of social desirability bias, whereby respondents may have overstated their compliance. Subgroup analyses (e.g., postgraduate livestock producers, low-knowledge veterinarians) should be interpreted with caution due to small numbers. Finally, the analysis was restricted to survey variables and did not incorporate observational or biological data. Despite these challenges, the sample size, geographic spread, and methodological consistency across the two countries can provide robust evidence to guide policy and training.

## 5 Conclusion

This study demonstrates that knowledge, education, and perceptions shape biosecurity and PPE practices among livestock producers and veterinarians in Armenia and Moldova, and that there are still substantial risks associated with limited awareness of zoonotic risks and inconsistent use of PPE in both groups. These findings underscore the need for targeted, accessible, context-specific training which emphasizes the risks of zoonotic transmission and the importance and correct use of PPE, in addition to affordable PPE provision. In parallel, strengthening animal health and zoonotic disease control programs that improve the safety of animal-derived products could further reduce exposure risks for both producers and consumers. Strengthening such measures is essential to reducing zoonotic transmission in both agricultural and veterinary settings.

## Data Availability

The raw data supporting the conclusions of this article will be made available by the authors, without undue reservation.
