# Supplementary material for: Personal biosecurity among livestock producers and veterinarians in Armenia and the Republic of Moldova
Source: Front Vet Sci. 2026 May 8;13:1784276. doi: 10.3389/fvets.2026.1784276 (PMC13196288; doi:10.3389/fvets.2026.1784276)
Supplement: Supplementary file 1 [file Data_Sheet_1.docx]

**SUPPLEMENTARY FILE 1**

**Personal Biosecurity Questionnaire for Farmers**

**Introduction**

This questionnaire is intended for the person at the farm who has more contact with the animals.

**Please read aloud to the interviewee**:

*“This questionnaire will take approximately 20 minutes to complete. It covers farm characteristics, farmer/employee characteristics, diseases, general practices on the farm and implementation of personal protective measures and understanding of the concept.*

*We would like to understand the practices that you implement on the farm to protect yourself from getting infected with diseases from animals and why do you decide to implement them or not.*

*Most of the questions are single or multiple choice. Please answer all the questions.*

*Before we start, please confirm that you have read and signed the consent form.*

*Please note that your answers to this questionnaire will be anonymous.”*

| Name/ID of the interviewer: Date of survey: |
| --- |
| Location: Province: District: Village:  Language of the interview: *(single choice)*  □ Moldovan □ Russian □ Other (please specify) |

**A. Farm characteristics**

1. What type(s) of livestock and production system do you have on your farm and what is the current herd size (taking into account the adult animals only)? Select all that apply. *(multiple choice)*

| Type of livestock | Type of production system | Herd size |
| --- | --- | --- |
| Beef cattle | - Nomadic/transhumance - Always kept indoor - Extensive - Semi-extensive - Other (please specify)… |  |
| Dairy cattle | - Always kept indoor - Extensive - Semi-extensive - Other (please specify)… |  |
| Cattle of mixed purpose | - Always kept indoor - Extensive - Semi-extensive - Other (please specify)… |  |
| Sheep | - Nomadic/transhumance - Always kept indoor - Extensive - Semi-extensive - Other (please specify)… |  |
| Goat | - Nomadic/transhumance - Always kept indoor - Extensive - Semi-extensive - Other (please specify)… |  |

**B. Socio-demographic features**

2. What is your age? *(single choice)*

| □ 15-20 | □ 21-30 | □ 31-40 | □ 41-50 | □ ≥51 |
| --- | --- | --- | --- | --- |

3. What is your gender? *(single choice)*

| □ Man | □ Woman | □ I prefer not to answer |
| --- | --- | --- |

4. What is your marital status? *(single choice)*

| □ Married | □ Not married □ Divorced □ Widowed | □ I prefer not to answer |
| --- | --- | --- |

5. What is your highest level of education? *(single choice)*

| □ Illiterate | □ Primary school | □ Middle school |
| --- | --- | --- |
| □ High school | □ University degree | □ Postgraduate degree |

5.1. If you have a university degree, is your education related to agriculture? *(single choice)*

| □ Yes | □ No |
| --- | --- |

6. What is your role on the farm? *(single choice)*

| □ Farm owner | □ Salaried employee | □ Other (please specify) ……… |
| --- | --- | --- |

7. How long have you been handling livestock? *(single choice)*

| □ ≤5 years | □ 6-10 years | □ 11-20 years | □ ≥21 years |
| --- | --- | --- | --- |

8. How many people (apart from you) work on the farm? *(single choice)*

| □ 0 | □ 1-5 | □ 6-10 | □ ≥11 |
| --- | --- | --- | --- |

**C. Zoonoses**

9. Do you think it is possible for you to get a disease from your animals? *(single choice)*

| □ Yes | □ No | □ I do not know |
| --- | --- | --- |

9.1. If yes, which diseases do you know can be transmitted from animals to humans? Select all that apply. *(multiple choice)*

| - Anthrax - Brucellosis - Ovine chlamydiosis - Ringworm - Animal tuberculosis - Ecthyma contagiosum (Orf) - Crimean Congo Haemorrhagic fever - Cryptosporidiosis - Leptospirosis - Cowpox - Echinococcosis - Q Fever |  |
| --- | --- |
| - Other, please specify…………………………………………………………………………………………………………………. |  |

10. Which of the following diseases have been diagnosed in your farm or region? Select all that apply. *(multiple choice)*

| - Anthrax - Brucellosis - Ovine chlamydiosis - Ringworm - Animal tuberculosis - Ecthyma contagiosum (Orf) - Crimean Congo Haemorrhagic fever - Cryptosporidiosis - Leptospirosis - Cowpox - Echinococcosis - Q Fever |
| --- |
| □ Other, please specify……………………………………………………………………………………………………………………….   - None - I do not know |

11. Have you or anyone working in your farm got a disease from the animals in the last 10 years? *(single choice)*

| □ Yes | □ No | □ I do not know |
| --- | --- | --- |

11.1. If yes, how many times in the last 10 years? *(single choice)*

| □ 1 | □ 2 | □ 3 | □ >3 |
| --- | --- | --- | --- |

11.2. If yes, what diseases? Please write them down. *(free text) ……………………………………………………..*

**D. Practices**

12. How often do you clean/disinfect your farm? *(single choice)*

| □ Daily | □ Once a week | □ Every other week | □ Monthly | □ Several times a year | □ Once a year |
| --- | --- | --- | --- | --- | --- |

13. How often do you clean/disinfect commonly used farm equipment? *(single choice)*

| □ After every use | □ Daily | □ Once a week | □ Once a year |
| --- | --- | --- | --- |
| □ Every other week | □ Monthly | □ Several times a year |  |

14. Do you have…? *(all items; single choice for each item)*

| Item | Yes | No | Yes, but I do not use it |
| --- | --- | --- | --- |
| A changing room on your farm | □ | □ | □ |
| A sink with |  |  |  |
| running water | □ | □ | □ |
| a bar of soap | □ | □ | □ |
| a liquid/foam soap dispenser | □ | □ | □ |
| alcohol-based disinfectants | □ | □ | □ |
| disposable towels | □ | □ | □ |
| hand dryer | □ | □ | □ |

15. How often do you wash your farm dedicated clothing? *(single choice)*

| □ Daily | □ Weekly | □ Monthly | □ Rarely | □ Never |
| --- | --- | --- | --- | --- |

16. When you wash your farm dedicated clothing, do you wash them separately from your other clothes? *(single choice)*

| □ Yes | □ No |
| --- | --- |

17. Please indicate how frequently do you apply following practices: *(all measures; single choice for each measure)*

| Practices | Always | Sometimes | Never |
| --- | --- | --- | --- |
| Wash hands before contact with animals | □ | □ | □ |
| Wash hands after contact with body fluids of animals (e.g., blood, abortion materials, foetus, etc.) | □ | □ | □ |
| If gloves are used, wash hands after contact with animals | □ | □ | □ |
| Wash hands before eating, drinking and smoking | □ | □ | □ |
| Wash the wound site after getting a cut or scratch at the farm | □ | □ | □ |
| Cover cuts or abrasions on your skin with waterproof bandages | □ | □ | □ |
| Boil or pasteurize milk before consumption | □ | □ | □ |
| Sell milk or cheese made from unpasteurized milk? | □ | □ | □ |
| Wash fruits and vegetables thoroughly before eating or cooking | □ | □ | □ |
| Consume raw or undercooked meat | □ | □ | □ |
| Dispose animal waste or dead animals in nearby fields | □ | □ | □ |
| Ensure a clean and safe water supply for both animals and human consumption | □ | □ | □ |
| Regularly apply antiparasitic medicines to pets | □ | □ | □ |
| Not allow pets on the farm | □ | □ | □ |
| Not feed pets with viscera | □ | □ | □ |

18. Please indicate how frequently do you implement the following practices: *(all practices; single choice for each practice)*

| Practices | Always | Sometimes | Never |
| --- | --- | --- | --- |
| Isolate sick animals | □ | □ | □ |
| Seek veterinary advice promptly if signs of illness are observed | □ | □ | □ |
| Treat infected animals promptly with appropriate medications as recommended by a veterinarian | □ | □ | □ |
| Vaccinate animals against diseases transmissible to humans, e.g. brucellosis and anthrax | □ | □ | □ |

**E. Use of personal protective equipment:**

19. Which personal protective equipment do you use during the following situations? *(all situations; multiple choice for practices)*

| Situations | Farm-dedicated overalls | Farm-dedicated boots | Gloves | Face masks | Protective glasses |
| --- | --- | --- | --- | --- | --- |
| Contact with healthy animals (e.g. during hoof trimming, shearing, assisting vet for vaccinating animals, etc.) |  |  |  |  |  |
| Contact with clinically sick animals |  |  |  |  |  |
| Contact with animals suspected of having a disease |  |  |  |  |  |
| Contact with dead animals and disposal of carcasses |  |  |  |  |  |
| Assisting parturition |  |  |  |  |  |
| Disposal of aborted placenta and stillbirths |  |  |  |  |  |
| Cleaning surfaces/  stables |  |  |  |  |  |

**F. Perceived risk of contracting a disease from animals**

20. In your view, how likely can you get a disease from an animal when the following situations are carried out on the farm? *(all practices; single choice for each practice)*

| **Situations** | **Very likely** | **Likely** | **Unlikely** |
| --- | --- | --- | --- |
| Contact with healthy animals | □ | □ | □ |
| Contact with clinically sick animals | □ | □ | □ |
| Contact with an animal suspected of having a disease | □ | □ | □ |
| Contact with dead animals and disposal of carcasses | □ | □ | □ |
| Assisting parturition | □ | □ | □ |
| Disposal of aborted foetal membranes and stillbirths | □ | □ | □ |
| Cleaning surfaces/stables | □ | □ | □ |

**G. Motivators for and obstacles to PPE use**

21. Please indicate to what extent you agree with following statements related to implement or not to implement personal protection measures. *(all statements; single choice for each statement)*

| **I use personal protective equipment…** | **Strongly agree** | **Agree** | **Disagree** |
| --- | --- | --- | --- |
| to protect my health | □ | □ | □ |
| to protect the health of my family | □ | □ | □ |
| because I already have health problems that make me more vulnerable | □ | □ | □ |
| because I know of others who got infected from animals | □ | □ | □ |
| because the veterinarian advised me to do so | □ | □ | □ |
| because friends/colleagues convinced me that it is important | □ | □ | □ |
| to prevent introducing diseases into the farm | □ | □ | □ |
| because I know that the animals I work with are infected with diseases that could be transmitted to me | □ | □ | □ |
| to improve the health and welfare of the animals | □ | □ | □ |
| to improve the productivity of the animals | □ | □ | □ |
| because it is recommended by regulations | □ | □ | □ |
| **I do not use personal protective equipment to prevent getting infections from the animals because…** | **Strongly agree** | **Agree** | **Disagree** |
| it is expensive | □ | □ | □ |
| I do not know how and when to use | □ | □ | □ |
| it is difficult to use | □ | □ | □ |
| it is time consuming | □ | □ | □ |
| it is uncomfortable to use | □ | □ | □ |
| it is too hot or too humid | □ | □ | □ |

**H. Education and training**

22. Does anyone discuss personal protective measures with you to avoid diseases transmitted from animals? *(single choice for the first level (yes/no); multiple choice for Yes)*

| □ Yes, with… |
| --- |
| □ Official veterinarian at central level |
| □ Official regional level/field veterinarian |
| □ Private veterinarian |
| □ Livestock associations |
| - Extension officers or paravets |
| - Public health personnel (doctors, nurses…) |
| □ Other, please specify………………………………………………………………………………………………………………… |
| □ No |

22.1. If no, what might be the reasons? Select all that apply. *(multiple choice)*

| □ Veterinarians do not have enough time to discuss with farmers |
| --- |
| □ Veterinarians are not interested in discussing it with farmers |
| □ Veterinarians do not think that personal protective measures will be implemented by the farmers |
| □ Veterinarians may believe that farmers have lack of awareness on disease risks |
| □ Veterinarians may believe that farmers already know |
| □ Veterinarians may believe that farmers do not want to invest in personal protective measures |

23. Have you and your employees received training on diseases transmissible from animals to humans and/or how to prevent them? *(single choice)*

| □ Yes | □ No |
| --- | --- |

24. Are you interested in learning more about diseases transmissible from animals to humans and/or how to prevent them? *(single choice)*

| □ Yes | □ No |
| --- | --- |

24.1. If yes, what format would you prefer to learn more and by whom?

| Format. Select all that apply *(multiple choice)* | | | |
| --- | --- | --- | --- |
| □ Face-to-face | □ Workshop | □ Online | □ Leaflets or brochures |
| □ Short videos | □ TV |  |  |
| 24.2 By whom. Select all that apply *(multiple choice)* | | | |
| □ Official veterinarian at central level | □ Official regional level/field veterinarian | □ Private veterinarian | □ Livestock associations |
| □ Extension officers or paravets | □ Public health personnel (doctors, nurses…) | | □ Academicians |

25. Is there anything else you would like to add? *(free text)*

……………………………………………………………………………………………………………………………………………………………

Thank you for completing the survey!

**Personal Biosecurity Questionnaire for Veterinarians**

**Introduction**

This questionnaire is intended for veterinarians who work with farm animals, i.e. ruminants.

**Please read aloud to the interviewee**:

*“This questionnaire will take approximately 20 minutes to complete. It covers demographic features, zoonoses, and understanding of personal biosecurity and its implementation on farms.*

*We would like to understand the practices that you implement on the farm to protect yourself from getting infected with zoonoses and why do you decide to implement them or not.*

*Most of the questions are single or multiple choice. Please answer all the questions.*

*Before we start, please confirm that you have read and signed the consent form.*

*Please note that your answers to this questionnaire will be anonymous.”*

| Name/ID of the interviewer: Date of survey: |
| --- |
| Location: Province: District: Village:  Language of the interview: *(single choice)*  □ Moldovan □ Russian □ Other (please specify) |

**A. Socio-demographic features**

1. What is your age? *(single choice)*

| □ 18-30 | □ 31-40 | □ 41-50 | □ ≥51 |
| --- | --- | --- | --- |

2. What is your gender? *(single choice)*

| □ Male | □ Female | □ I prefer not to answer |
| --- | --- | --- |

3. What is your marital status? *(single choice)*

| - Married | - Not married □ Divorced □ Widowed | - I do not want to answer |
| --- | --- | --- |

4. How many years of experience do you have in the veterinary field? *(single choice)*

| □ ≤10 | □ 11-20 | □ 21-30 | □ 31-40 | □ ≥41 |
| --- | --- | --- | --- | --- |

5. What type(s) of livestock do you work with in the field? Select all that apply. *(multiple choice)*

| □ Beef cattle | □ Dairy cattle | □ Sheep | □ Goat |
| --- | --- | --- | --- |

6. How many days per a week do you visit ruminant farms? *(single choice)*

| □ 1-2 | □ 3-4 | □ Daily |  |
| --- | --- | --- | --- |

**B. Zoonoses**

7. What is your level of knowledge on zoonoses? Enter a number in a scale from 1 to 10, where 10 is the highest level of knowledge *(number from 1 to 10)*

8. Which of the following diseases can be transmitted from animals to humans? Select all that apply. *(multiple choice)*

| - Anthrax - Brucellosis - Ovine chlamydiosis - Ringworm - Animal tuberculosis - Ecthyma contagiosum (Orf) - Crimean Congo Haemorrhagic fever - Cryptosporidiosis - Leptospirosis - Cowpox - Echinococcosis - Q Fever |
| --- |
| □ Other, please specify…………………………………………………………………………………………………………………….. |

9. Have you got infected with any zoonotic diseases in the last 10 years? *(single choice)*

| □ Yes | □ No |
| --- | --- |

9.1. If yes, which disease(s)? Select all that apply. *(multiple choice)*

| - Anthrax - Brucellosis - Ovine chlamydiosis - Ringworm - Animal tuberculosis - Ecthyma contagiosum (Orf) - Crimean Congo haemorrhagic fever - Cryptosporidiosis - Leptospirosis - Cowpox - Echinococcosis - Q Fever |  |  |  |
| --- | --- | --- | --- |
| □ Other, please specify…………………………………………………………………………………………………… | | | |

9.2. If yes, how many times in the last 10 years? *(single choice)*

| □ 1 | □ 2 | □ 3 | □ ≥4 |  |
| --- | --- | --- | --- | --- |
| **C. Practices**  10. How often do you wash your farm dedicated clothing? *(single choice)*   \| □ Daily \| □ Weekly \| □ Monthly \| □ Rarely \| □ Never \| \| --- \| --- \| --- \| --- \| --- \|   11. Where do you wash your farm dedicated clothing? *(single choice)*   \| □ At home \| □ At workplace \| \| --- \| --- \|   12. When you wash your farm dedicated clothing, do you wash them separately from your other clothes? *(single choice)*   \| □ Yes \| □ No \| \| --- \| --- \|   13. Please indicate how frequently do you apply following practices. *(all measures; single choice for each measure)*   \| Practices \| Always \| Sometimes \| Never \| \| --- \| --- \| --- \| --- \| \| Wash hands before contact with animals \| □ \| □ \| □ \| \| Wash hands after contact with animals and their body fluids (e.g. blood, abortion materials, foetus, etc.) \| □ \| □ \| □ \| \| If gloves are used, wash hands after contact with animals \| □ \| □ \| □ \| \| Wash hands after touching equipment contaminated with the body fluids of animals \| □ \| □ \| □ \| \| Wash hands after removing personal protective equipment \| □ \| □ \| □ \| \| Wash hands with bar soap \| □ \| □ \| □ \| \| Wash hands with liquid/foam soap \| □ \| □ \| □ \| \| Wash hands before eating, drinking and smoking \| □ \| □ \| □ \| \| Use alcohol-based disinfectants after washing hands \| □ \| □ \| □ \| \| Use disposable towels to dry hands \| □ \| □ \| □ \| \| Wash the wound site after getting a cut or scratch at the farm \| □ \| □ \| □ \| \| Cover cuts or abrasions on your skin with waterproof bandages \| □ \| □ \| □ \| \| Dispose sharps (e.g., needles, etc.) in sharps containers \| □ \| □ \| □ \| \| Remove the needle from the syringe by hand \| □ \| □ \| □ \| \| Remove the needle cap with mouth \| □ \| □ \| □ \|   14. How often do you implement personal protective equipment during the following situations? *(all situations; multiple choice for practices)*   \| Situations \| Overalls \| Gloves \| Face masks \| Protective glasses \| \| --- \| --- \| --- \| --- \| --- \| \| Physical examination of healthy animals \|  \|  \|  \|  \| \| Physical examination of clinically sick animals \|  \|  \|  \|  \| \| Physical examination of an animal suspected of having an infectious disease \|  \|  \|  \|  \| \| Post-mortem examination \|  \|  \|  \|  \| \| Contact with blood, body substances, membranes, faeces or fluids of animals \|  \|  \|  \|  \| \| Surgery \|  \|  \|  \|  \| \| Vaccination \|  \|  \|  \|  \| \| Blood sampling \|  \|  \|  \|  \| \| Treatment (e.g. injections) \|  \|  \|  \|  \| \| Parturition \|  \|  \|  \|  \| \| Examination and disposal of aborted foetal membranes and stillbirths \|  \|  \|  \|  \| \| Disposal of carcasses \|  \|  \|  \|  \|   **D. Perceived risk of contracting zoonoses**  15. What is the likelihood of exposure to zoonoses when the following situations are carried out on farms? *(all practices; single choice for each practice)*   \| Situations \| Very likely \| Likely \| Unlikely \| \| --- \| --- \| --- \| --- \| \| Physical examination of healthy animals \| □ \| □ \| □ \| \| Physical examination of clinically sick animals \| □ \| □ \| □ \| \| Physical examination of an animal suspected of having an infectious disease \| □ \| □ \| □ \| \| Post-mortem examination \| □ \| □ \| □ \| \| When contact with blood, body substances, membranes, faeces, or fluids of animals \| □ \| □ \| □ \| \| Surgery \| □ \| □ \| □ \| \| Vaccination \| □ \| □ \| □ \| \| Blood sampling \| □ \| □ \| □ \| \| Treatment (e.g. injections) \| □ \| □ \| □ \| \| Vaccination \| □ \| □ \| □ \| \| Parturition \| □ \| □ \| □ \| \| Examination and disposal of aborted foetal membranes and stillbirths \| □ \| □ \| □ \| \| Disposal of carcasses \| □ \| □ \| □ \|   **E. Motivators for and obstacles to PPE use**  16. Please indicate to what extent you agree with following statements related to implement or not to implement personal protection measures. *(all statements; single choice for each statement)*   \| **I implement personal protective equipment** \| **Strongly agree** \| **Agree** \| **Disagree** \| \| --- \| --- \| --- \| --- \| \| to protect my health \| □ \| □ \| □ \| \| to protect the health of my family/colleagues/friends/relatives \| □ \| □ \| □ \| \| because I already have health problems that make me more vulnerable \| □ \| □ \| □ \| \| because of a previous zoonotic disease experience \| □ \| □ \| □ \| \| because my colleagues convinced me that it is important \| □ \| □ \| □ \| \| to prevent introducing/spreading diseases into other farms \| □ \| □ \| □ \| \| because I think implementing personal biosecurity is an individual responsibility \| □ \| □ \| □ \| \| to improve the health and welfare of the animals \| □ \| □ \| □ \| \| to improve the productivity of the animals \| □ \| □ \| □ \| \| because it is recommended by regulations \| □ \| □ \| □ \| \| because farmers expect me to implement personal biosecurity on their farms \| □ \| □ \| □ \| \| because using PPE allows me to feel protected from diseases \| □ \| □ \| □ \| \| because using PPE keeps my clothes clean \| □ \| □ \| □ \| \| **I do not implement personal protective equipment to prevent zoonoses because…** \| **Strongly agree** \| **Agree** \| **Disagree** \| \| I think implementing personal biosecurity is a requirement of farmers \| □ \| □ \| □ \| \| there are negative perceptions from farmers when I use PPE \| □ \| □ \| □ \| \| my colleagues think I am being too cautious when implementing personal biosecurity \| □ \| □ \| □ \| \| When PPE is provided by the farmer, it is often visibly dirty \| □ \| □ \| □ \| \| When PPE is provided by the farmer, the size often does not fit me \| □ \| □ \| □ \| \| it is expensive \| □ \| □ \| □ \| \| I do not know how and when to use \| □ \| □ \| □ \| \| it is difficult to use \| □ \| □ \| □ \| \| it is time consuming \| □ \| □ \| □ \| \| it is uncomfortable to use \| □ \| □ \| □ \| \| it is too hot or too humid \| □ \| □ \| □ \| | | | | |
|  | | | | |

**F. Education and training**

17. Do you advise the farmer(s)/employee(s) on personal biosecurity to protect themselves from zoonoses? *(single choice)*

| □ Yes | □ No |
| --- | --- |

17.1. If no, what might be the reasons? Select all that apply. *(multiple choice)*

| □ Farmers are not interested in discussing |
| --- |
| □ Farmers do not want to invest in personal biosecurity |
| □ Farmers do not have the economic power to invest in personal biosecurity |
| □ Farmers do not have time to implement personal biosecurity |
| □ Farmers are not aware of infectious disease risks |

18. Have you received training on zoonoses and/or how to prevent them after graduation from the vet school? *(single choice)*

| □ Yes | □ No |
| --- | --- |

18.1 If yes, what diseases were covered? *(free text)*

……………………………………………………………………………………………………

19. Are you interested in learning more about zoonoses and/or how to prevent them? *(single choice)*

| □ Yes | □ No |
| --- | --- |

19.1. If yes, what format would you prefer to learn more and by whom?

| a. Format. Select all that apply *(multiple choice)* | | | |
| --- | --- | --- | --- |
| □ Face-to-face | □ Workshop | □ Online | □ Leaflets or brochures |
| □ Short videos | □ TV |  |  |
| b. By whom. Select all that apply *(multiple choice)* | | | |
| □ Official veterinarian at central level | □ Official veterinarian at field level | □ Private veterinarian | □ Livestock associations |
| □ Extension officers or paravets | □ Public health personnel (doctors, nurses…) | | □ Academics |

20. Is there anything else you would like to add? *(free text)*

……………………………………………………………………………………………………………………………………………………………

Thank you for completing the survey!
