# Supplementary material for: Personal biosecurity among livestock producers and veterinarians in Armenia and the Republic of Moldova
Source: Front Vet Sci. 2026 May 8;13:1784276. doi: 10.3389/fvets.2026.1784276 (PMC13196288; doi:10.3389/fvets.2026.1784276)
Supplement: Supplementary file 3 [file Data_Sheet_3.docx]

```{r setup, include=FALSE}

knitr::opts_chunk$set(echo = TRUE)

```

```{r, echo = FALSE, warning= FALSE, message =FALSE}

library(dplyr)

library(readxl)

library(writexl)

library(kableExtra)

library(ggplot2)

library(ggthemes)

library(tidyverse)

library(tidyr)

library(FactoMineR)

library(factoextra)

library(cluster)

library(fpc)

library(reshape2)

library(purrr)

library(ggrepel)

library(janitor)

library(openxlsx)

FARM <- read_xlsx("FARMS_BIOSEC.xlsx")

VETS <- read_xlsx("VETS_BIOSEC.xlsx")

```

#########################################

# FARMERS

#########################################

# 29/10/2025 ANALYSIS FARMER BIOSEC

```{r}

# FARM <- FARM[,-c(4:8)] semi-extensive, transhumance...

# FARM <- FARM[,-c(2)] district

# FARM <- FARM[,-c(6)] repeated

# FARM <- FARM[,-c(7)] NAs - degree in agriculture

# FARM <- FARM[,-c(7)] almost all are farm owners

# FARM <- FARM[,-c(10)] NAs

# FARM <- FARM[,-c(10)] NAs

# FARM <- FARM[,-c(41,47,53,59,65,71,77)] NAs

#colnames(FARM)[3] <- "age"

#colnames(FARM)[4] <- "gender"

#colnames(FARM)[5] <- "marital status"

#colnames(FARM)[6] <- "education"

#colnames(FARM)[7] <- "time_livestock"

#colnames(FARM)[8] <- "workers"

#colnames(FARM)[2] <- "production_type"

#colnames(FARM)[9] <- "perception"

# ============================

# 2️⃣ Define supplementary variables

# ============================

non_active_vars <- c("Country", "gender", "perception", "education",

"production_type", "age", "marital status", "time_livestock", "workers")

# FARM 760 OBSERVATIONS AND 42 VARIABLES

FARM_BIOSEC <- FARM[,c(1:9,11:40)]

# ============================================================

# FARM BIOSECURITY MCA PIPELINE

# ============================================================

# ============================================================

# FILTERING — AUTOMATED + SUMMARY REPORT

# ============================================================

all_vars <- names(FARM_BIOSEC)

active_candidates <- setdiff(all_vars, non_active_vars)

filter_summary <- data.frame(

Variable = character(),

Reason_Removed = character(),

stringsAsFactors = FALSE

)

# --- Remove variables with >15% missing ---

missing_prop <- sapply(FARM_BIOSEC[active_candidates], function(x) mean(is.na(x)))

to_remove_missing <- names(missing_prop[missing_prop > 0.15])

if (length(to_remove_missing) > 0) {

filter_summary <- rbind(filter_summary,

data.frame(Variable = to_remove_missing,

Reason_Removed = ">15% missing"))

}

active_candidates <- setdiff(active_candidates, to_remove_missing)

# --- Remove variables with <10% non-missing observations ---

obs_prop <- sapply(FARM_BIOSEC[active_candidates], function(x) mean(!is.na(x)))

to_remove_low_obs <- names(obs_prop[obs_prop < 0.10])

if (length(to_remove_low_obs) > 0) {

filter_summary <- rbind(filter_summary,

data.frame(Variable = to_remove_low_obs,

Reason_Removed = "<10% non-missing"))

}

active_candidates <- setdiff(active_candidates, to_remove_low_obs)

# --- Robustness check: ≥20 (or ≥15) observations per category ---

keep_vars <- c()

for (v in active_candidates) {

counts <- table(FARM_BIOSEC[[v]])

if (all(counts >= 20)) {

keep_vars <- c(keep_vars, v)

} else if (all(counts >= 15)) {

keep_vars <- c(keep_vars, v)

message(paste("Variable", v, "accepted with relaxed threshold (≥15 per category)"))

} else {

filter_summary <- rbind(filter_summary,

data.frame(Variable = v,

Reason_Removed = "<15 obs in ≥1 category"))

}

}

active_candidates <- keep_vars

# --- Remove variables with >12 categories ---

to_remove_too_many_levels <- sapply(active_candidates, function(v) {

length(unique(na.omit(FARM_BIOSEC[[v]]))) > 12

})

if (any(to_remove_too_many_levels)) {

removed <- active_candidates[to_remove_too_many_levels]

filter_summary <- rbind(filter_summary,

data.frame(Variable = removed,

Reason_Removed = ">12 categories"))

active_candidates <- active_candidates[!to_remove_too_many_levels]

}

# ============================================================

# BUILD MCA DATASET

# ============================================================

FARM_BIOSEC_mca <- FARM_BIOSEC[, c(non_active_vars[1:9], active_candidates)]

# Remove variables with ≥90% identical answers

vars_to_remove <- names(FARM_BIOSEC_mca)[sapply(FARM_BIOSEC_mca, function(col) {

col <- col[!is.na(col)]

if (length(col) == 0) return(FALSE)

max(prop.table(table(col))) >= 0.9

})]

if (length(vars_to_remove) > 0) {

filter_summary <- rbind(filter_summary,

data.frame(Variable = vars_to_remove,

Reason_Removed = "≥90% identical answers"))

}

FARM_BIOSEC_mca <- FARM_BIOSEC_mca %>% select(-all_of(vars_to_remove))

# Recalculate valid active variables

valid_active_vars <- setdiff(names(FARM_BIOSEC_mca), non_active_vars)

# Convert to factors

FARM_BIOSEC_mca[, valid_active_vars] <- lapply(FARM_BIOSEC_mca[, valid_active_vars], as.factor)

# --- Print summary of filtering ---

cat("\n============================\nFILTER SUMMARY\n============================\n")

if (nrow(filter_summary) > 0) {

print(filter_summary)

write_xlsx(filter_summary, "Filter_Summary_BIOSEC.xlsx")

} else {

cat("✅ No variables removed during filtering.\n")

}

cat("\nFinal active variables (", length(valid_active_vars), "):\n")

print(valid_active_vars)

# ============================================================

# MCA ANALYSIS

# ============================================================

supp_vars <- c(1:9, (ncol(FARM_BIOSEC_mca)-2):ncol(FARM_BIOSEC_mca))

mca_result <- MCA(FARM_BIOSEC_mca, quali.sup = supp_vars, graph = FALSE)

# ============================================================

# ETA² + V-TEST

# ============================================================

compute_eta2 <- function(mca_obj, data, dims = 2) {

res <- data.frame(Variable = character(), Dimension = integer(), Eta2 = numeric())

for (var in names(data)) {

if (!is.factor(data[[var]])) next

for (d in 1:dims) {

dim_coords <- mca_obj$ind$coord[, d]

group_means <- tapply(dim_coords, data[[var]], mean)

group_sizes <- table(data[[var]])

overall_mean <- mean(dim_coords)

ss_between <- sum(group_sizes * (group_means - overall_mean)^2)

ss_total <- sum((dim_coords - overall_mean)^2)

res <- rbind(res, data.frame(Variable = var, Dimension = d, Eta2 = ss_between / ss_total))

}

}

return(res)

}

eta2_table <- compute_eta2(mca_result, FARM_BIOSEC_mca[, valid_active_vars], dims = 2)

eta2_table$Eta2 <- round(eta2_table$Eta2, 3)

write_xlsx(eta2_table, "eta2_BIOSEC.xlsx")

dimdesc_res <- dimdesc(mca_result, axes = c(1, 2), proba = 1)

extract_vtest_table_matrix <- function(dimdesc_res, dim_num) {

cat_mat <- dimdesc_res[[dim_num]]$category

levels <- rownames(cat_mat)

data.frame(Level = levels,

Vtest = cat_mat[, "Estimate"],

P_value = cat_mat[, "p.value"],

Dimension = dim_num,

stringsAsFactors = FALSE)

}

dim1_df <- extract_vtest_table_matrix(dimdesc_res, 1)

dim2_df <- extract_vtest_table_matrix(dimdesc_res, 2)

vtest_table <- rbind(dim1_df, dim2_df)

vtest_table$Variable <- sub("=.*", "", vtest_table$Level)

final_table <- merge(vtest_table, eta2_table, by = c("Variable", "Dimension"), all.x = TRUE)

write_xlsx(final_table, "final_table_MCA_BIOSEC.xlsx")

# ============================================================

# MCA BIPLOTS BY CLUSTER VARIABLE

# ============================================================

eig_vals <- mca_result$eig

dim1_pct <- round(eig_vals[1, "percentage of variance"], 1)

dim2_pct <- round(eig_vals[2, "percentage of variance"], 1)

top_var_levels <- final_table %>%

filter(P_value < 0.05) %>%

group_by(Dimension) %>%

arrange(desc(abs(Vtest))) %>%

slice_head(n = 3) %>%

ungroup()

var_coords <- as.data.frame(mca_result$var$coord)

var_coords$Level <- rownames(var_coords)

var_coords$Variable <- sub("=.*", "", var_coords$Level)

var_coords$Dimension <- ifelse(

var_coords$Level %in% top_var_levels$Level[top_var_levels$Dimension == 1], 1,

ifelse(var_coords$Level %in% top_var_levels$Level[top_var_levels$Dimension == 2], 2, NA)

)

var_coords$Label <- ifelse(!is.na(var_coords$Dimension), var_coords$Level, NA)

caption_text <- paste("Non-active variables:", paste(non_active_vars, collapse = ", "))

# --- Function to generate and save cluster biplot ---

make_cluster_biplot <- function(cluster_var) {

plot_data <- as.data.frame(mca_result$ind$coord)

plot_data$Cluster <- FARM_BIOSEC_mca[[cluster_var]]

p <- ggplot() +

geom_point(data = plot_data,

aes(x = `Dim 1`, y = `Dim 2`, color = Cluster),

alpha = 0.6, size = 2) +

geom_segment(data = var_coords[!is.na(var_coords$Dimension), ],

aes(x = 0, y = 0, xend = `Dim 1`, yend = `Dim 2`, color = factor(Dimension)),

arrow = arrow(length = unit(0.02, "npc"))) +

geom_text(data = var_coords[!is.na(var_coords$Dimension), ],

aes(x = `Dim 1`, y = `Dim 2`, label = Label, color = factor(Dimension)),

vjust = -0.5, fontface = "bold", size = 3.5) +

labs(

title = paste("MCA Biplot Colored by", cluster_var),

x = paste0("Dimension 1 (", dim1_pct, "%)"),

y = paste0("Dimension 2 (", dim2_pct, "%)"),

caption = caption_text

) +

theme_minimal() +

theme(

plot.title = element_text(face = "bold", size = 14),

plot.caption = element_text(hjust = 0, size = 9, face = "italic"),

legend.position = "right"

)

# Save high-resolution PDF

filename <- paste0("MCA_Biplot_", cluster_var, "_HighRes.pdf")

ggsave(filename, plot = p, device = cairo_pdf, width = 12, height = 10, dpi = 300, units = "in")

cat(paste0("\n✅ Saved: ", filename, " (300 dpi)\n"))

}

# Generate three cluster plots

make_cluster_biplot("education")

make_cluster_biplot("Country")

make_cluster_biplot("perception")

cat("\n✅ All three MCA biplots with clusters saved (PDF, 300 dpi).\n")

cat(" - MCA_Biplot_education_HighRes.pdf\n")

cat(" - MCA_Biplot_Country_HighRes.pdf\n")

cat(" - MCA_Biplot_perception_HighRes.pdf\n")

# ============================================================

# END OF PIPELINE

# ============================================================

cat("\n✅ MCA pipeline completed successfully.\n")

# ============================================================

# SUMMARY TABLES — BY COUNTRY, EDUCATION, PERCEPTION

# ============================================================

# Helper function to compute frequency + percentage tables

summarize_by_group <- function(data, group_var, active_vars) {

summary_list <- list()

for (v in active_vars) {

tab <- data %>%

group_by(!!sym(group_var), !!sym(v)) %>%

summarise(N = n(), .groups = "drop") %>%

group_by(!!sym(group_var)) %>%

mutate(Percent = round(100 * N / sum(N), 1)) %>%

rename(Level = !!sym(v))

tab$Variable <- v

summary_list[[v]] <- tab

}

bind_rows(summary_list)

}

# Run summaries

summary_country <- summarize_by_group(FARM_BIOSEC_mca, "Country", valid_active_vars)

summary_education <- summarize_by_group(FARM_BIOSEC_mca, "education", valid_active_vars)

summary_perception <- summarize_by_group(FARM_BIOSEC_mca, "perception", valid_active_vars)

# Save all tables to a single Excel workbook

wb <- createWorkbook()

addWorksheet(wb, "By_Country")

addWorksheet(wb, "By_Education")

addWorksheet(wb, "By_Perception")

writeData(wb, "By_Country", summary_country)

writeData(wb, "By_Education", summary_education)

writeData(wb, "By_Perception", summary_perception)

saveWorkbook(wb, "Summary_Tables_BIOSEC.xlsx", overwrite = TRUE)

cat("\n✅ Summary tables created and saved to 'Summary_Tables_BIOSEC.xlsx'\n")

cat(" Sheets:\n - By_Country\n - By_Education\n - By_Perception\n")

```

# 29/10/2025 ANALYSIS FARMER PPE

```{r}

# ============================

# Define supplementary variables

# ============================

non_active_vars <- c("Country", "gender", "perception", "education",

"production_type", "age", "marital status", "time_livestock", "workers")

# FARM 760 OBSERVATIONS AND 42 VARIABLES

FARM_PPE <- FARM[,c(1:9,41:99)]

# ============================================================

# FILTERING — AUTOMATED + SUMMARY REPORT

# ============================================================

all_vars <- names(FARM_PPE)

active_candidates <- setdiff(all_vars, non_active_vars)

filter_summary <- data.frame(

Variable = character(),

Reason_Removed = character(),

stringsAsFactors = FALSE

)

# --- Remove variables with >15% missing ---

missing_prop <- sapply(FARM_PPE[active_candidates], function(x) mean(is.na(x)))

to_remove_missing <- names(missing_prop[missing_prop > 0.15])

if (length(to_remove_missing) > 0) {

filter_summary <- rbind(filter_summary,

data.frame(Variable = to_remove_missing,

Reason_Removed = ">15% missing"))

}

active_candidates <- setdiff(active_candidates, to_remove_missing)

# --- Remove variables with <10% non-missing observations ---

obs_prop <- sapply(FARM_PPE[active_candidates], function(x) mean(!is.na(x)))

to_remove_low_obs <- names(obs_prop[obs_prop < 0.10])

if (length(to_remove_low_obs) > 0) {

filter_summary <- rbind(filter_summary,

data.frame(Variable = to_remove_low_obs,

Reason_Removed = "<10% non-missing"))

}

active_candidates <- setdiff(active_candidates, to_remove_low_obs)

# --- Robustness check: ≥20 (or ≥15) observations per category ---

keep_vars <- c()

for (v in active_candidates) {

counts <- table(FARM_PPE[[v]])

if (all(counts >= 20)) {

keep_vars <- c(keep_vars, v)

} else if (all(counts >= 15)) {

keep_vars <- c(keep_vars, v)

message(paste("Variable", v, "accepted with relaxed threshold (≥15 per category)"))

} else {

filter_summary <- rbind(filter_summary,

data.frame(Variable = v,

Reason_Removed = "<15 obs in ≥1 category"))

}

}

active_candidates <- keep_vars

# --- Remove variables with >12 categories ---

to_remove_too_many_levels <- sapply(active_candidates, function(v) {

length(unique(na.omit(FARM_PPE[[v]]))) > 12

})

if (any(to_remove_too_many_levels)) {

removed <- active_candidates[to_remove_too_many_levels]

filter_summary <- rbind(filter_summary,

data.frame(Variable = removed,

Reason_Removed = ">12 categories"))

active_candidates <- active_candidates[!to_remove_too_many_levels]

}

# ============================================================

# BUILD MCA DATASET

# ============================================================

FARM_PPE_mca <- FARM_PPE[, c(non_active_vars[1:9], active_candidates)]

# Remove variables with ≥90% identical answers

vars_to_remove <- names(FARM_PPE_mca)[sapply(FARM_PPE_mca, function(col) {

col <- col[!is.na(col)]

if (length(col) == 0) return(FALSE)

max(prop.table(table(col))) >= 0.9

})]

if (length(vars_to_remove) > 0) {

filter_summary <- rbind(filter_summary,

data.frame(Variable = vars_to_remove,

Reason_Removed = "≥90% identical answers"))

}

FARM_PPE_mca <- FARM_PPE_mca %>% select(-all_of(vars_to_remove))

# Recalculate valid active variables

valid_active_vars <- setdiff(names(FARM_PPE_mca), non_active_vars)

# Convert to factors

FARM_PPE_mca[, valid_active_vars] <- lapply(FARM_PPE_mca[, valid_active_vars], as.factor)

# --- Print summary of filtering ---

cat("\n============================\nFILTER SUMMARY\n============================\n")

if (nrow(filter_summary) > 0) {

print(filter_summary)

write_xlsx(filter_summary, "Filter_Summary_PPE.xlsx")

} else {

cat("✅ No variables removed during filtering.\n")

}

cat("\nFinal active variables (", length(valid_active_vars), "):\n")

print(valid_active_vars)

# ============================================================

# MCA ANALYSIS

# ============================================================

supp_vars <- c(1:9, (ncol(FARM_PPE_mca)-2):ncol(FARM_PPE_mca))

mca_result <- MCA(FARM_PPE_mca, quali.sup = supp_vars, graph = FALSE)

# ============================================================

# ETA² + V-TEST

# ============================================================

compute_eta2 <- function(mca_obj, data, dims = 2) {

res <- data.frame(Variable = character(), Dimension = integer(), Eta2 = numeric())

for (var in names(data)) {

if (!is.factor(data[[var]])) next

for (d in 1:dims) {

dim_coords <- mca_obj$ind$coord[, d]

group_means <- tapply(dim_coords, data[[var]], mean)

group_sizes <- table(data[[var]])

overall_mean <- mean(dim_coords)

ss_between <- sum(group_sizes * (group_means - overall_mean)^2)

ss_total <- sum((dim_coords - overall_mean)^2)

res <- rbind(res, data.frame(Variable = var, Dimension = d, Eta2 = ss_between / ss_total))

}

}

return(res)

}

eta2_table <- compute_eta2(mca_result, FARM_PPE_mca[, valid_active_vars], dims = 2)

eta2_table$Eta2 <- round(eta2_table$Eta2, 3)

write_xlsx(eta2_table, "eta2_PPE.xlsx")

dimdesc_res <- dimdesc(mca_result, axes = c(1, 2), proba = 1)

extract_vtest_table_matrix <- function(dimdesc_res, dim_num) {

cat_mat <- dimdesc_res[[dim_num]]$category

levels <- rownames(cat_mat)

data.frame(Level = levels,

Vtest = cat_mat[, "Estimate"],

P_value = cat_mat[, "p.value"],

Dimension = dim_num,

stringsAsFactors = FALSE)

}

dim1_df <- extract_vtest_table_matrix(dimdesc_res, 1)

dim2_df <- extract_vtest_table_matrix(dimdesc_res, 2)

vtest_table <- rbind(dim1_df, dim2_df)

vtest_table$Variable <- sub("=.*", "", vtest_table$Level)

final_table <- merge(vtest_table, eta2_table, by = c("Variable", "Dimension"), all.x = TRUE)

write_xlsx(final_table, "final_table_MCA_PPE.xlsx")

# ============================================================

# MCA BIPLOTS BY CLUSTER VARIABLE

# ============================================================

eig_vals <- mca_result$eig

dim1_pct <- round(eig_vals[1, "percentage of variance"], 1)

dim2_pct <- round(eig_vals[2, "percentage of variance"], 1)

top_var_levels <- final_table %>%

filter(P_value < 0.05) %>%

group_by(Dimension) %>%

arrange(desc(abs(Vtest))) %>%

slice_head(n = 3) %>%

ungroup()

var_coords <- as.data.frame(mca_result$var$coord)

var_coords$Level <- rownames(var_coords)

var_coords$Variable <- sub("=.*", "", var_coords$Level)

var_coords$Dimension <- ifelse(

var_coords$Level %in% top_var_levels$Level[top_var_levels$Dimension == 1], 1,

ifelse(var_coords$Level %in% top_var_levels$Level[top_var_levels$Dimension == 2], 2, NA)

)

var_coords$Label <- ifelse(!is.na(var_coords$Dimension), var_coords$Level, NA)

caption_text <- paste("Non-active variables:", paste(non_active_vars, collapse = ", "))

# --- Function to generate and save cluster biplot ---

make_cluster_biplot <- function(cluster_var) {

plot_data <- as.data.frame(mca_result$ind$coord)

plot_data$Cluster <- FARM_PPE_mca[[cluster_var]]

p <- ggplot() +

geom_point(data = plot_data,

aes(x = `Dim 1`, y = `Dim 2`, color = Cluster),

alpha = 0.6, size = 2) +

geom_segment(data = var_coords[!is.na(var_coords$Dimension), ],

aes(x = 0, y = 0, xend = `Dim 1`, yend = `Dim 2`, color = factor(Dimension)),

arrow = arrow(length = unit(0.02, "npc"))) +

geom_text(data = var_coords[!is.na(var_coords$Dimension), ],

aes(x = `Dim 1`, y = `Dim 2`, label = Label, color = factor(Dimension)),

vjust = -0.5, fontface = "bold", size = 3.5) +

labs(

title = paste("MCA Biplot Colored by", cluster_var),

x = paste0("Dimension 1 (", dim1_pct, "%)"),

y = paste0("Dimension 2 (", dim2_pct, "%)"),

caption = caption_text

) +

theme_minimal() +

theme(

plot.title = element_text(face = "bold", size = 14),

plot.caption = element_text(hjust = 0, size = 9, face = "italic"),

legend.position = "right"

)

# Save high-resolution PDF

filename <- paste0("MCA_Biplot_", cluster_var, "_HighRes.pdf")

ggsave(filename, plot = p, device = cairo_pdf, width = 12, height = 10, dpi = 300, units = "in")

cat(paste0("\n✅ Saved: ", filename, " (300 dpi)\n"))

}

# Generate three cluster plots

make_cluster_biplot("education")

make_cluster_biplot("Country")

make_cluster_biplot("perception")

cat("\n✅ All three MCA biplots with clusters saved (PDF, 300 dpi).\n")

cat(" - MCA_Biplot_education_HighRes.pdf\n")

cat(" - MCA_Biplot_Country_HighRes.pdf\n")

cat(" - MCA_Biplot_perception_HighRes.pdf\n")

# ============================================================

# END OF PIPELINE

# ============================================================

cat("\n✅ MCA pipeline completed successfully.\n")

library(janitor)

library(openxlsx)

library(dplyr)

library(tidyr)

# Helper function to compute frequency + percentage tables

summarize_by_group <- function(data, group_var, active_vars) {

summary_list <- list()

for (v in active_vars) {

tab <- data %>%

group_by(!!sym(group_var), !!sym(v)) %>%

summarise(N = n(), .groups = "drop") %>%

group_by(!!sym(group_var)) %>%

mutate(Percent = round(100 * N / sum(N), 1)) %>%

rename(Level = !!sym(v))

tab$Variable <- v

summary_list[[v]] <- tab

}

bind_rows(summary_list)

}

# Run summaries

summary_country <- summarize_by_group(FARM_PPE_mca, "Country", valid_active_vars)

summary_education <- summarize_by_group(FARM_PPE_mca, "education", valid_active_vars)

summary_perception <- summarize_by_group(FARM_PPE_mca, "perception", valid_active_vars)

# Save all tables to a single Excel workbook

wb <- createWorkbook()

addWorksheet(wb, "By_Country")

addWorksheet(wb, "By_Education")

addWorksheet(wb, "By_Perception")

writeData(wb, "By_Country", summary_country)

writeData(wb, "By_Education", summary_education)

writeData(wb, "By_Perception", summary_perception)

saveWorkbook(wb, "Summary_Tables_PPE.xlsx", overwrite = TRUE)

cat("\n✅ Summary tables created and saved to 'Summary_Tables_PPE.xlsx'\n")

cat(" Sheets:\n - By_Country\n - By_Education\n - By_Perception\n")

```

# 29/10/2025 ANALYSIS VET BIOSEC

```{r}

# ============================================================

# FARM BIOSECURITY MCA PIPELINE — CLEAN, ROBUST & PUBLISHABLE

# ============================================================

VETS$KNOWLEDGE_ZOONOSIS_2 <- ifelse(VETS$Knowledge_zoonosis <=4,"1-4",

ifelse(VETS$Knowledge_zoonosis >=5 & VETS$Knowledge_zoonosis <=8,"5-8","9-10"))

non_active_vars <- c("Country", "gender", "Beef cattle", "Dairy cattle", "Sheep", "Goat","age",

"marital_status", "years_experience", "Days_per_week_on_farm",

"KNOWLEDGE_ZOONOSIS_2")

VETS_BIOSEC <- VETS[, c(1:10,13:30,120)]

# ============================================================

# FILTERING — AUTOMATED + SUMMARY REPORT

# ============================================================

set.seed(123)

# ============================================================

# ADAPTIVE FILTERING

# ============================================================

all_vars <- names(VETS_BIOSEC)

active_candidates <- setdiff(all_vars, non_active_vars)

filter_variables <- function(data, candidates, missing_thresh = 0.4, min_cat_count = 2) {

filter_summary <- data.frame(Variable = character(), Reason_Removed = character(), stringsAsFactors = FALSE)

# Too much missing

missing_prop <- sapply(data[candidates], function(x) mean(is.na(x)))

to_remove_missing <- names(missing_prop[missing_prop > missing_thresh])

if (length(to_remove_missing) > 0) {

filter_summary <- rbind(filter_summary,

data.frame(Variable = to_remove_missing,

Reason_Removed = paste0(">", missing_thresh*100, "% missing")))

}

candidates <- setdiff(candidates, to_remove_missing)

# ≥90% identical

vars_identical <- names(data[candidates])[sapply(data[candidates], function(col) {

col <- col[!is.na(col)]

if (length(col) == 0) return(FALSE)

max(prop.table(table(col))) >= 0.9

})]

if (length(vars_identical) > 0) {

filter_summary <- rbind(filter_summary,

data.frame(Variable = vars_identical, Reason_Removed = "≥90% identical answers"))

}

candidates <- setdiff(candidates, vars_identical)

# Too many categories

too_many_levels <- sapply(candidates, function(v) length(unique(na.omit(data[[v]]))) > 12)

if (any(too_many_levels)) {

removed <- candidates[too_many_levels]

filter_summary <- rbind(filter_summary,

data.frame(Variable = removed, Reason_Removed = ">12 categories"))

candidates <- candidates[!too_many_levels]

}

# Minimum observations per category

keep_vars <- c()

for (v in candidates) {

counts <- table(data[[v]])

if (all(counts >= min_cat_count)) keep_vars <- c(keep_vars, v)

else filter_summary <- rbind(filter_summary,

data.frame(Variable = v, Reason_Removed = paste0("<", min_cat_count, " obs in ≥1 category")))

}

candidates <- keep_vars

return(list(vars = candidates, summary = filter_summary))

}

# Adaptive thresholds

min_cats <- 2

miss_thresh <- 0.4

repeat {

res <- filter_variables(VETS_BIOSEC, active_candidates, miss_thresh, min_cats)

active_candidates <- res$vars

filter_summary <- res$summary

if (length(active_candidates) >= 2) break

miss_thresh <- miss_thresh + 0.1

min_cats <- max(1, min_cats - 1)

message(paste("⚠️ Relaxing thresholds: missing <", miss_thresh, "| min_cat_count =", min_cats))

if (miss_thresh >= 0.8 && min_cats <= 1) stop("❌ Unable to find ≥2 active variables.")

}

# ============================================================

# BUILD MCA DATASET

# ============================================================

VETS_BIOSEC_mca <- VETS_BIOSEC[, c(intersect(non_active_vars, names(VETS_BIOSEC)), active_candidates)]

valid_active_vars <- setdiff(names(VETS_BIOSEC_mca), non_active_vars)

VETS_BIOSEC_mca[, valid_active_vars] <- lapply(VETS_BIOSEC_mca[, valid_active_vars], as.factor)

# ============================================================

# RUN MCA

# ============================================================

supp_vars <- intersect(non_active_vars, names(VETS_BIOSEC_mca))

mca_result <- MCA(VETS_BIOSEC_mca, quali.sup = supp_vars, graph = FALSE)

# ============================================================

# ETA² TABLE

# ============================================================

compute_eta2 <- function(mca_obj, data, dims = 2) {

dims <- min(dims, ncol(mca_obj$ind$coord))

res <- data.frame(Variable = character(), Dimension = integer(), Eta2 = numeric())

for (var in names(data)) {

if (!is.factor(data[[var]])) next

for (d in 1:dims) {

dim_coords <- mca_obj$ind$coord[, d]

group_means <- tapply(dim_coords, data[[var]], mean)

group_sizes <- table(data[[var]])

overall_mean <- mean(dim_coords)

ss_between <- sum(group_sizes * (group_means - overall_mean)^2)

ss_total <- sum((dim_coords - overall_mean)^2)

res <- rbind(res, data.frame(Variable = var, Dimension = d, Eta2 = ss_between / ss_total))

}

}

return(res)

}

eta2_table <- compute_eta2(mca_result, VETS_BIOSEC_mca[, valid_active_vars], dims = 2)

eta2_table$Eta2 <- round(eta2_table$Eta2, 3)

write_xlsx(eta2_table, "Eta2_Table_BIOSEC.xlsx")

# ============================================================

# SAFE V-TEST TABLE

# ============================================================

safe_dimdesc_extract <- function(mca_res, proba = 1) {

dd <- dimdesc(mca_res, axes = c(1,2), proba = proba)

out <- data.frame()

for (dim_num in c(1,2)) {

cat_mat <- dd[[dim_num]]$category

if (!is.null(cat_mat) && nrow(cat_mat) > 0) {

tmp <- data.frame(Level = rownames(cat_mat),

Vtest = cat_mat[, "Estimate"],

P_value = cat_mat[, "p.value"],

Dimension = dim_num,

stringsAsFactors = FALSE)

out <- rbind(out, tmp)

}

}

return(out)

}

vtest_table <- safe_dimdesc_extract(mca_result)

# ============================================================

# MCA BIPLOTS (PDF, 200 DPI)

# ============================================================

dim1_pct <- round(mca_result$eig[1,"percentage of variance"],1)

dim2_pct <- round(mca_result$eig[2,"percentage of variance"],1)

caption_text <- paste("Non-active variables:", paste(non_active_vars, collapse = ", "))

make_mca_biplot <- function(cluster_var) {

if (!(cluster_var %in% names(VETS_BIOSEC_mca))) {

warning(paste("Variable", cluster_var, "not found. Skipping plot."))

return(NULL)

}

plot_data <- as.data.frame(mca_result$ind$coord)

plot_data$Cluster <- VETS_BIOSEC_mca[[cluster_var]]

var_coords <- as.data.frame(mca_result$var$coord)

var_coords$Level <- rownames(var_coords)

var_coords$Variable <- sub("=.*", "", var_coords$Level)

var_coords$Label <- var_coords$Level # top contributors can be filtered

p <- ggplot() +

geom_point(data = plot_data, aes(x = `Dim 1`, y = `Dim 2`, color = Cluster),

alpha=0.6, size=2) +

# geom_segment(data = var_coords, aes(x=0, y=0, xend=`Dim 1`, yend=`Dim 2`),

# arrow=arrow(length=unit(0.02,"npc")), color="darkgrey") +

#geom_text_repel(data = var_coords, aes(x=`Dim 1`, y=`Dim 2`, label=Label),

# size=3, fontface="bold", color="black") +

labs(title=paste("MCA Biplot – Cluster by", cluster_var),

x=paste0("Dim 1 (", dim1_pct,"% variance)"),

y=paste0("Dim 2 (", dim2_pct,"% variance)"),

caption=caption_text) +

theme_minimal() +

theme(plot.title=element_text(face="bold", size=14),

plot.caption=element_text(face="italic", size=9),

legend.position="right")

pdf_file <- paste0("MCA_Biplot_", cluster_var, "_300dpi.pdf")

ggsave(pdf_file, plot=p, device=cairo_pdf, width=12, height=10, units="in", dpi=300)

cat(paste0("\n✅ Saved: ", pdf_file, "\n"))

}

# Generate biplots

make_mca_biplot("Country")

make_mca_biplot("KNOWLEDGE_ZOONOSIS_2")

# ============================================================

# SAVE FILTER SUMMARY & REPORT

# ============================================================

write_xlsx(filter_summary, "Filter_Summary_BIOSEC.xlsx")

cat("\n============================\nFILTER SUMMARY\n============================\n")

print(filter_summary)

cat("\nFinal active variables (", length(valid_active_vars), "):\n")

print(valid_active_vars)

cat("\n✅ MCA pipeline completed. Biplots saved in PDF (200 dpi).\n")

# Helper function to compute frequency + percentage tables

summarize_by_group <- function(data, group_var, active_vars) {

summary_list <- list()

for (v in active_vars) {

tab <- data %>%

group_by(!!sym(group_var), !!sym(v)) %>%

summarise(N = n(), .groups = "drop") %>%

group_by(!!sym(group_var)) %>%

mutate(Percent = round(100 * N / sum(N), 1)) %>%

rename(Level = !!sym(v))

tab$Variable <- v

summary_list[[v]] <- tab

}

bind_rows(summary_list)

}

# Run summaries

summary_country <- summarize_by_group(VETS_BIOSEC_mca, "Country", valid_active_vars)

summary_zoonosis <- summarize_by_group(VETS_BIOSEC_mca, "KNOWLEDGE_ZOONOSIS_2", valid_active_vars)

# Save all tables to a single Excel workbook

wb <- createWorkbook()

addWorksheet(wb, "By_Country")

addWorksheet(wb, "By_KNOWLEDGE_ZOONOSIS_2")

writeData(wb, "By_Country", summary_country)

writeData(wb, "By_KNOWLEDGE_ZOONOSIS_2", summary_zoonosis)

saveWorkbook(wb, "Summary_Tables_EBIOSEC.xlsx", overwrite = TRUE)

cat("\n✅ Summary tables created and saved to 'Summary_Tables_EBIOSEC.xlsx'\n")

cat(" Sheets:\n - By_Country\n - By_KNOWLEDGE_ZOONOSIS_2\n")

```

# 29/10/2025 ANALYSIS VET PPE

```{r}

VETS$KNOWLEDGE_ZOONOSIS_2 <- ifelse(VETS$Knowledge_zoonosis <=4,"1-4",

ifelse(VETS$Knowledge_zoonosis >=5 & VETS$Knowledge_zoonosis <=8,"5-8","9-10"))

non_active_vars <- c("Country", "gender", "Beef cattle", "Dairy cattle", "Sheep", "Goat","age",

"marital_status", "years_experience", "Days_per_week_on_farm",

"KNOWLEDGE_ZOONOSIS_2")

VETS_PPE <- VETS[, c(1:10,31:116,120)]

# ============================================================

# FILTERING — AUTOMATED + SUMMARY REPORT

# ============================================================

set.seed(123)

# ============================================================

# ADAPTIVE FILTERING

# ============================================================

all_vars <- names(VETS_PPE)

active_candidates <- setdiff(all_vars, non_active_vars)

filter_variables <- function(data, candidates, missing_thresh = 0.4, min_cat_count = 2) {

filter_summary <- data.frame(Variable = character(), Reason_Removed = character(), stringsAsFactors = FALSE)

# Too much missing

missing_prop <- sapply(data[candidates], function(x) mean(is.na(x)))

to_remove_missing <- names(missing_prop[missing_prop > missing_thresh])

if (length(to_remove_missing) > 0) {

filter_summary <- rbind(filter_summary,

data.frame(Variable = to_remove_missing,

Reason_Removed = paste0(">", missing_thresh*100, "% missing")))

}

candidates <- setdiff(candidates, to_remove_missing)

# ≥90% identical

vars_identical <- names(data[candidates])[sapply(data[candidates], function(col) {

col <- col[!is.na(col)]

if (length(col) == 0) return(FALSE)

max(prop.table(table(col))) >= 0.9

})]

if (length(vars_identical) > 0) {

filter_summary <- rbind(filter_summary,

data.frame(Variable = vars_identical, Reason_Removed = "≥90% identical answers"))

}

candidates <- setdiff(candidates, vars_identical)

# Too many categories

too_many_levels <- sapply(candidates, function(v) length(unique(na.omit(data[[v]]))) > 12)

if (any(too_many_levels)) {

removed <- candidates[too_many_levels]

filter_summary <- rbind(filter_summary,

data.frame(Variable = removed, Reason_Removed = ">12 categories"))

candidates <- candidates[!too_many_levels]

}

# Minimum observations per category

keep_vars <- c()

for (v in candidates) {

counts <- table(data[[v]])

if (all(counts >= min_cat_count)) keep_vars <- c(keep_vars, v)

else filter_summary <- rbind(filter_summary,

data.frame(Variable = v, Reason_Removed = paste0("<", min_cat_count, " obs in ≥1 category")))

}

candidates <- keep_vars

return(list(vars = candidates, summary = filter_summary))

}

# Adaptive thresholds

min_cats <- 2

miss_thresh <- 0.4

repeat {

res <- filter_variables(VETS_PPE, active_candidates, miss_thresh, min_cats)

active_candidates <- res$vars

filter_summary <- res$summary

if (length(active_candidates) >= 2) break

miss_thresh <- miss_thresh + 0.1

min_cats <- max(1, min_cats - 1)

message(paste("⚠️ Relaxing thresholds: missing <", miss_thresh, "| min_cat_count =", min_cats))

if (miss_thresh >= 0.8 && min_cats <= 1) stop("❌ Unable to find ≥2 active variables.")

}

# ============================================================

# BUILD MCA DATASET

# ============================================================

VETS_PPE_mca <- VETS_PPE[, c(intersect(non_active_vars, names(VETS_PPE)), active_candidates)]

valid_active_vars <- setdiff(names(VETS_PPE_mca), non_active_vars)

VETS_PPE_mca[, valid_active_vars] <- lapply(VETS_PPE_mca[, valid_active_vars], as.factor)

# ============================================================

# RUN MCA

# ============================================================

supp_vars <- intersect(non_active_vars, names(VETS_PPE_mca))

mca_result <- MCA(VETS_PPE_mca, quali.sup = supp_vars, graph = FALSE)

# ============================================================

# ETA² TABLE

# ============================================================

compute_eta2 <- function(mca_obj, data, dims = 2) {

dims <- min(dims, ncol(mca_obj$ind$coord))

res <- data.frame(Variable = character(), Dimension = integer(), Eta2 = numeric())

for (var in names(data)) {

if (!is.factor(data[[var]])) next

for (d in 1:dims) {

dim_coords <- mca_obj$ind$coord[, d]

group_means <- tapply(dim_coords, data[[var]], mean)

group_sizes <- table(data[[var]])

overall_mean <- mean(dim_coords)

ss_between <- sum(group_sizes * (group_means - overall_mean)^2)

ss_total <- sum((dim_coords - overall_mean)^2)

res <- rbind(res, data.frame(Variable = var, Dimension = d, Eta2 = ss_between / ss_total))

}

}

return(res)

}

eta2_table <- compute_eta2(mca_result, VETS_PPE_mca[, valid_active_vars], dims = 2)

eta2_table$Eta2 <- round(eta2_table$Eta2, 3)

write_xlsx(eta2_table, "Eta2_Table_PPE.xlsx")

# ============================================================

# SAFE V-TEST TABLE

# ============================================================

safe_dimdesc_extract <- function(mca_res, proba = 1) {

dd <- dimdesc(mca_res, axes = c(1,2), proba = proba)

out <- data.frame()

for (dim_num in c(1,2)) {

cat_mat <- dd[[dim_num]]$category

if (!is.null(cat_mat) && nrow(cat_mat) > 0) {

tmp <- data.frame(Level = rownames(cat_mat),

Vtest = cat_mat[, "Estimate"],

P_value = cat_mat[, "p.value"],

Dimension = dim_num,

stringsAsFactors = FALSE)

out <- rbind(out, tmp)

}

}

return(out)

}

vtest_table <- safe_dimdesc_extract(mca_result)

# ============================================================

# MCA BIPLOTS (PDF, 200 DPI)

# ============================================================

dim1_pct <- round(mca_result$eig[1,"percentage of variance"],1)

dim2_pct <- round(mca_result$eig[2,"percentage of variance"],1)

caption_text <- paste("Non-active variables:", paste(non_active_vars, collapse = ", "))

make_mca_biplot <- function(cluster_var) {

if (!(cluster_var %in% names(VETS_PPE_mca))) {

warning(paste("Variable", cluster_var, "not found. Skipping plot."))

return(NULL)

}

plot_data <- as.data.frame(mca_result$ind$coord)

plot_data$Cluster <- VETS_PPE_mca[[cluster_var]]

var_coords <- as.data.frame(mca_result$var$coord)

var_coords$Level <- rownames(var_coords)

var_coords$Variable <- sub("=.*", "", var_coords$Level)

var_coords$Label <- var_coords$Level # top contributors can be filtered

p <- ggplot() +

geom_point(data = plot_data, aes(x = `Dim 1`, y = `Dim 2`, color = Cluster),

alpha=0.6, size=2) +

# geom_segment(data = var_coords, aes(x=0, y=0, xend=`Dim 1`, yend=`Dim 2`),

# arrow=arrow(length=unit(0.02,"npc")), color="darkgrey") +

# geom_text_repel(data = var_coords, aes(x=`Dim 1`, y=`Dim 2`, label=Label),

# size=3, fontface="bold", color="black") +

labs(title=paste("MCA Biplot – Cluster by", cluster_var),

x=paste0("Dim 1 (", dim1_pct,"% variance)"),

y=paste0("Dim 2 (", dim2_pct,"% variance)"),

caption=caption_text) +

theme_minimal() +

theme(plot.title=element_text(face="bold", size=14),

plot.caption=element_text(face="italic", size=9),

legend.position="right")

pdf_file <- paste0("MCA_Biplot_", cluster_var, "_300dpi.pdf")

ggsave(pdf_file, plot=p, device=cairo_pdf, width=12, height=10, units="in", dpi=300)

cat(paste0("\n✅ Saved: ", pdf_file, "\n"))

}

# Generate biplots

make_mca_biplot("Country")

make_mca_biplot("KNOWLEDGE_ZOONOSIS_2")

# ============================================================

# SAVE FILTER SUMMARY & REPORT

# ============================================================

write_xlsx(filter_summary, "Filter_Summary_PPE.xlsx")

cat("\n============================\nFILTER SUMMARY\n============================\n")

print(filter_summary)

cat("\nFinal active variables (", length(valid_active_vars), "):\n")

print(valid_active_vars)

cat("\n✅ MCA pipeline completed. Biplots saved in PDF (200 dpi).\n")

# Helper function to compute frequency + percentage tables

summarize_by_group <- function(data, group_var, active_vars) {

summary_list <- list()

for (v in active_vars) {

tab <- data %>%

group_by(!!sym(group_var), !!sym(v)) %>%

summarise(N = n(), .groups = "drop") %>%

group_by(!!sym(group_var)) %>%

mutate(Percent = round(100 * N / sum(N), 1)) %>%

rename(Level = !!sym(v))

tab$Variable <- v

summary_list[[v]] <- tab

}

bind_rows(summary_list)

}

# Run summaries

summary_country <- summarize_by_group(VETS_PPE_mca, "Country", valid_active_vars)

summary_zoonosis <- summarize_by_group(VETS_PPE_mca, "KNOWLEDGE_ZOONOSIS_2", valid_active_vars)

# Save all tables to a single Excel workbook

wb <- createWorkbook()

addWorksheet(wb, "By_Country")

addWorksheet(wb, "By_KNOWLEDGE_ZOONOSIS_2")

writeData(wb, "By_Country", summary_country)

writeData(wb, "By_KNOWLEDGE_ZOONOSIS_2", summary_zoonosis)

saveWorkbook(wb, "Summary_Tables_EPPE.xlsx", overwrite = TRUE)

cat("\n✅ Summary tables created and saved to 'Summary_Tables_EPPE.xlsx'\n")

cat(" Sheets:\n - By_Country\n - By_KNOWLEDGE_ZOONOSIS_2\n")

```
