# Supplementary material for: Personal biosecurity among livestock producers and veterinarians in Armenia and the Republic of Moldova
Source: Front Vet Sci. 2026 May 8;13:1784276. doi: 10.3389/fvets.2026.1784276 (PMC13196288; doi:10.3389/fvets.2026.1784276)
Supplement: Supplementary file 4 [file Presentation_1.pptx]

## Slide 1
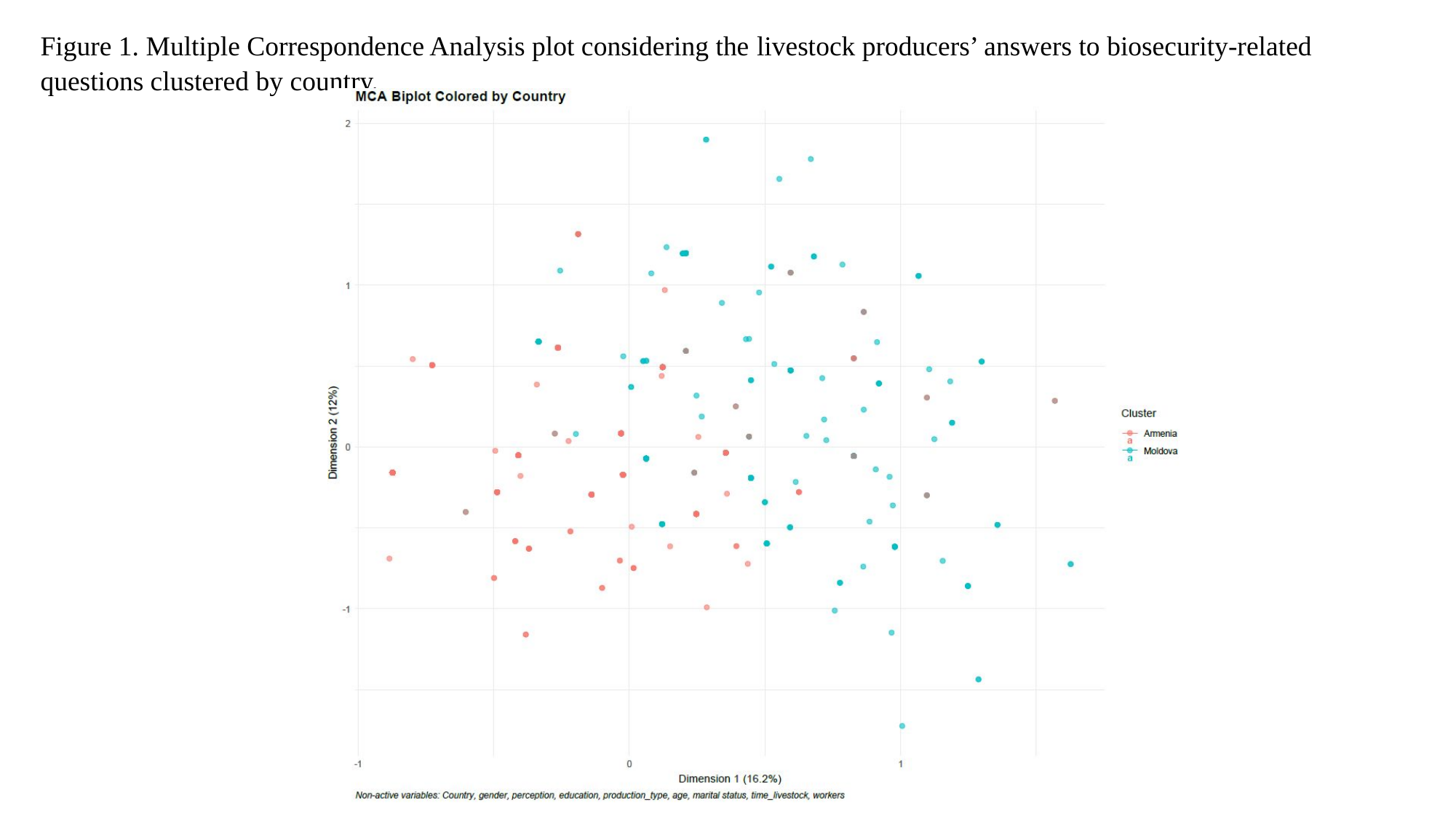

Figure 1. Multiple Correspondence Analysis plot considering the livestock producers’ answers to biosecurity-related questions clustered by country.

## Slide 2
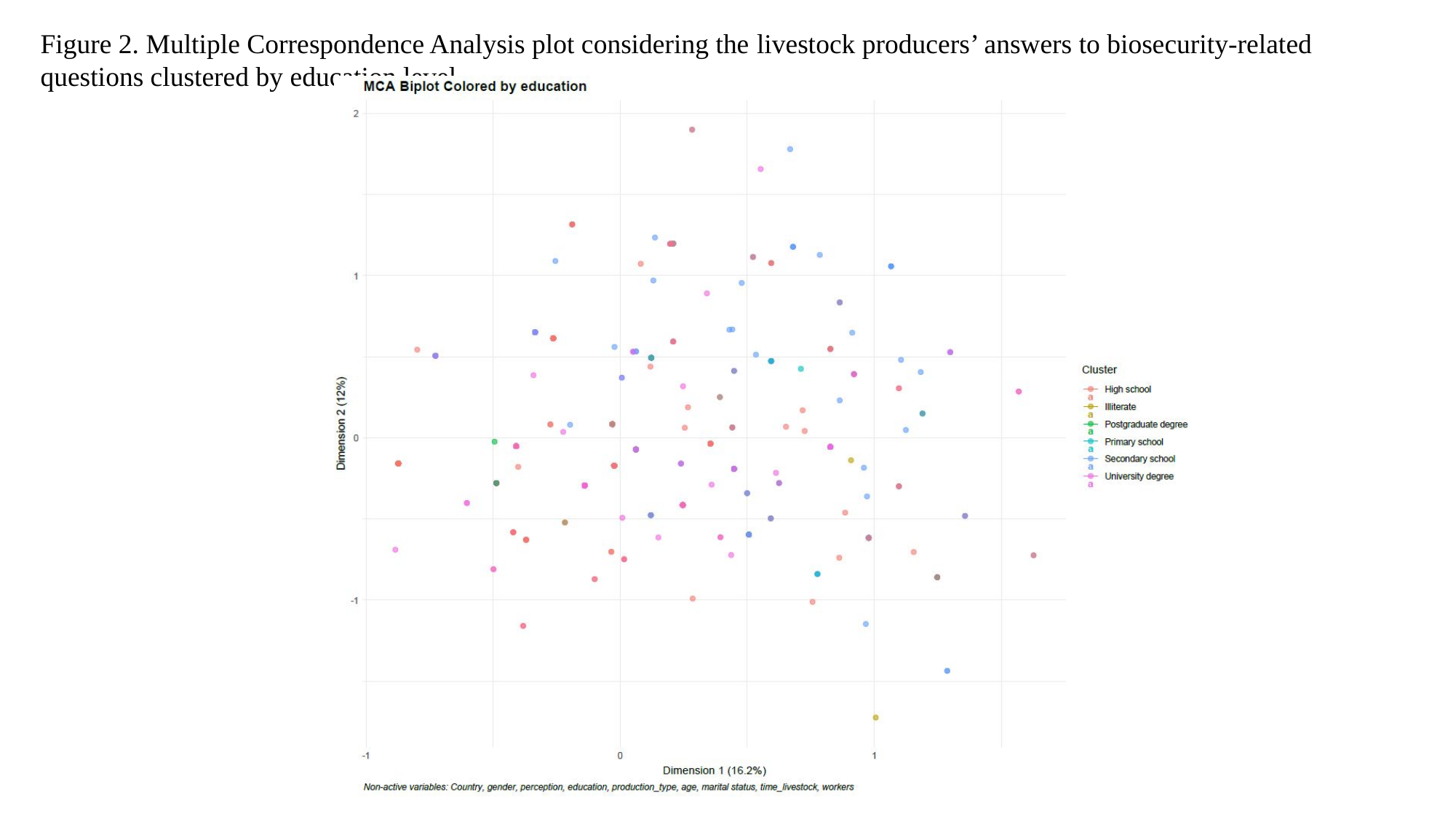

Figure 2. Multiple Correspondence Analysis plot considering the livestock producers’ answers to biosecurity-related questions clustered by education level.

## Slide 3
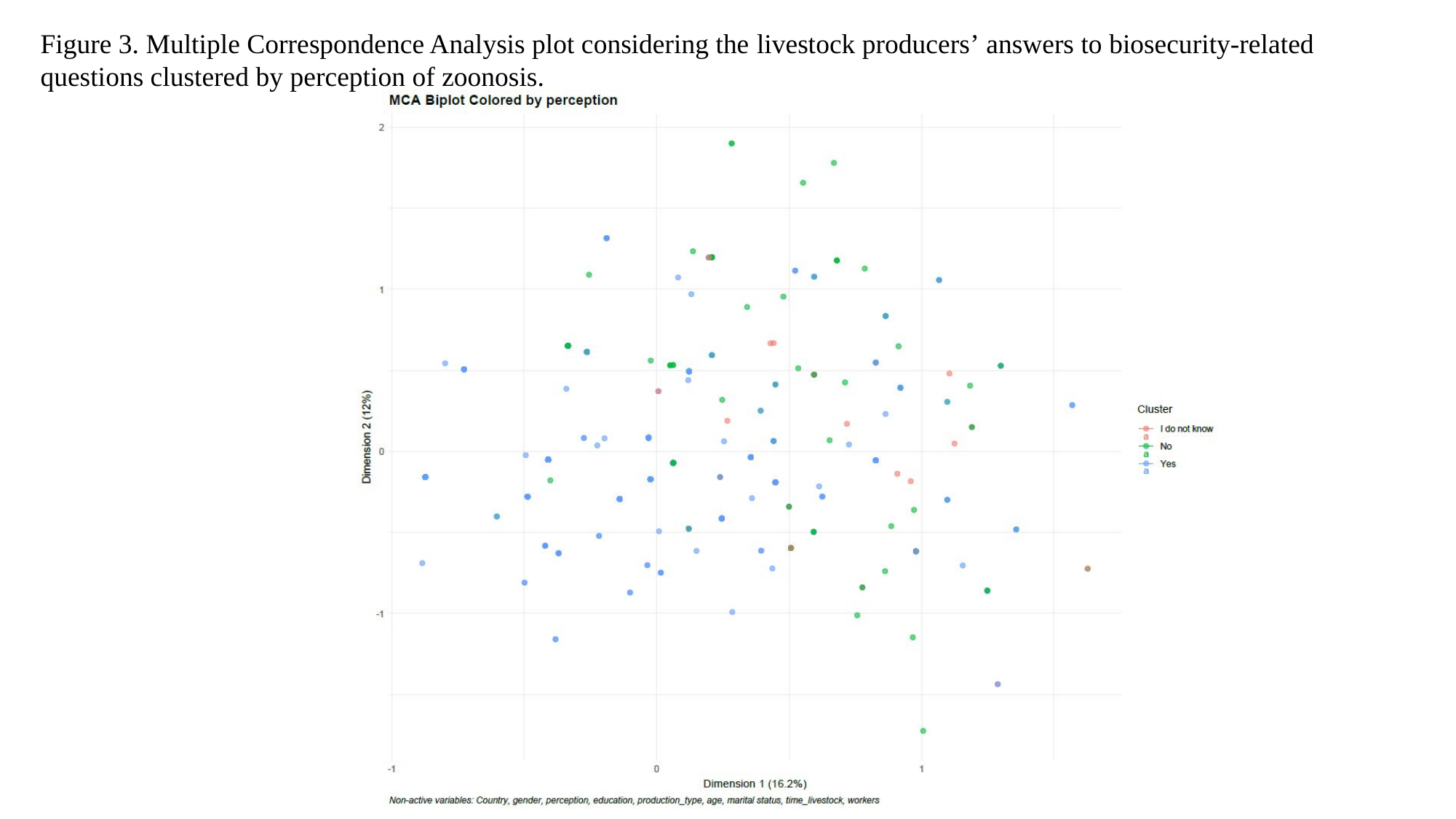

Figure 3. Multiple Correspondence Analysis plot considering the livestock producers’ answers to biosecurity-related questions clustered by perception of zoonosis.

## Slide 4
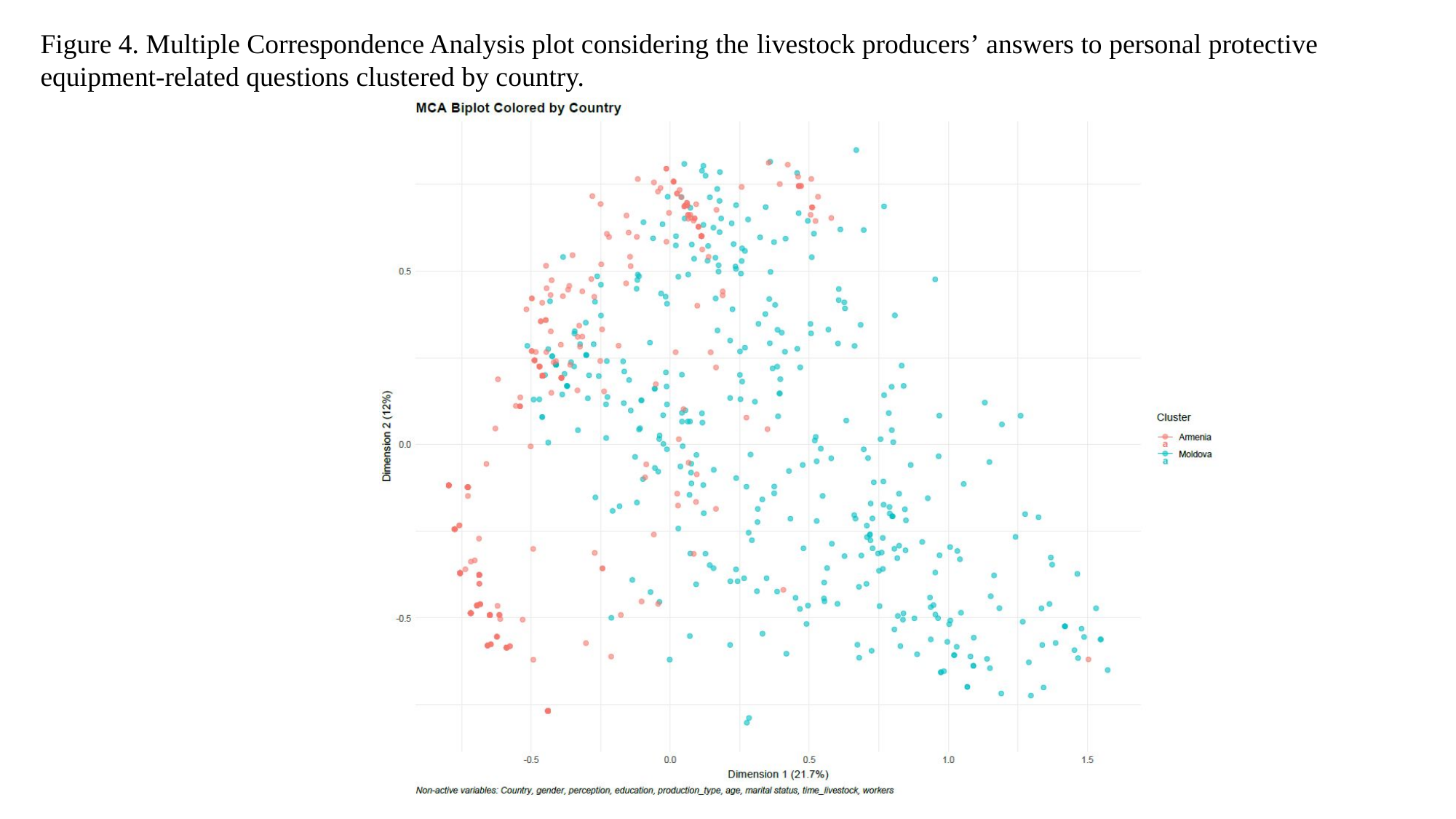

Figure 4. Multiple Correspondence Analysis plot considering the livestock producers’ answers to personal protective equipment-related questions clustered by country.

## Slide 5
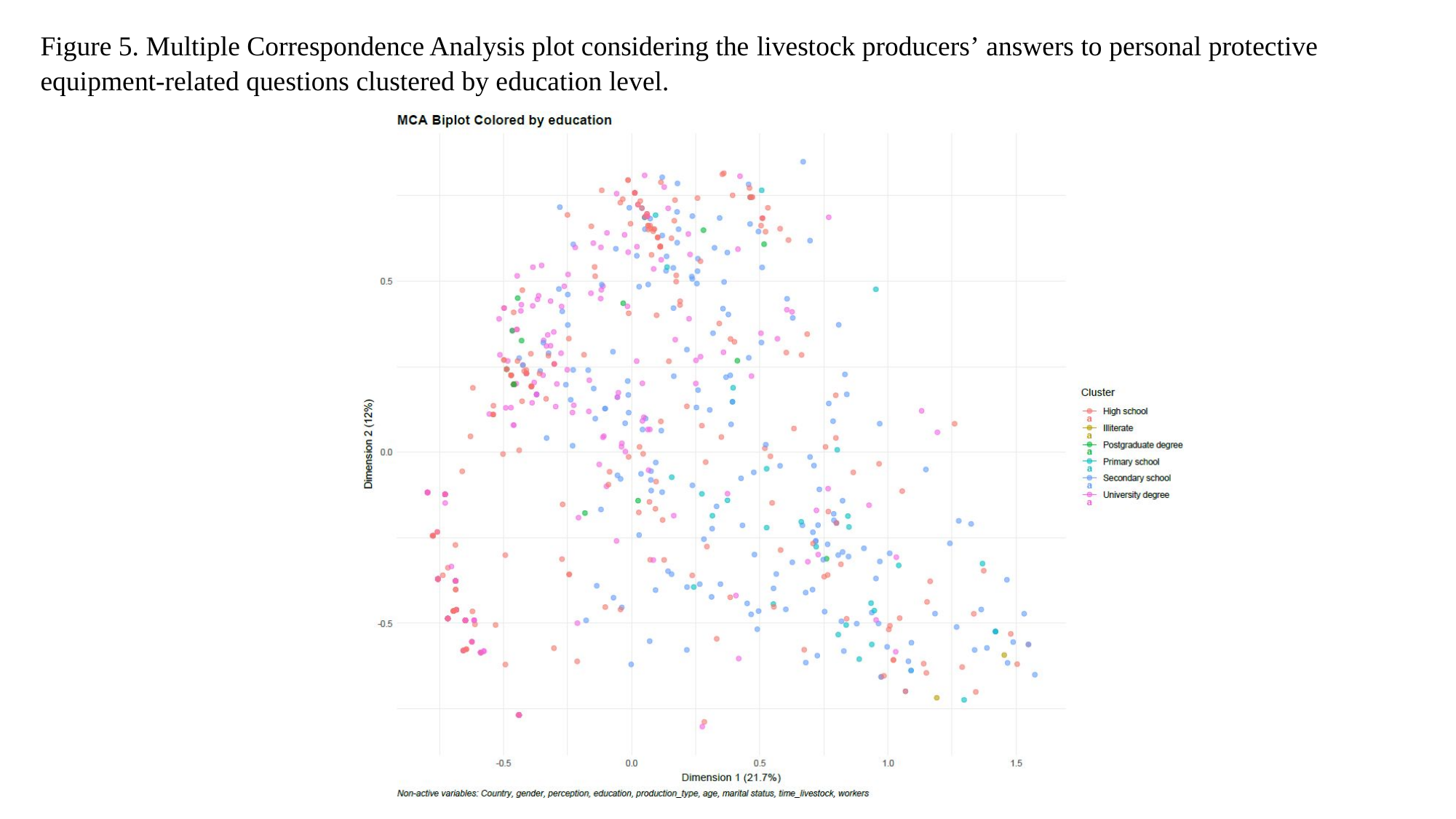

Figure 5. Multiple Correspondence Analysis plot considering the livestock producers’ answers to personal protective equipment-related questions clustered by education level.

## Slide 6
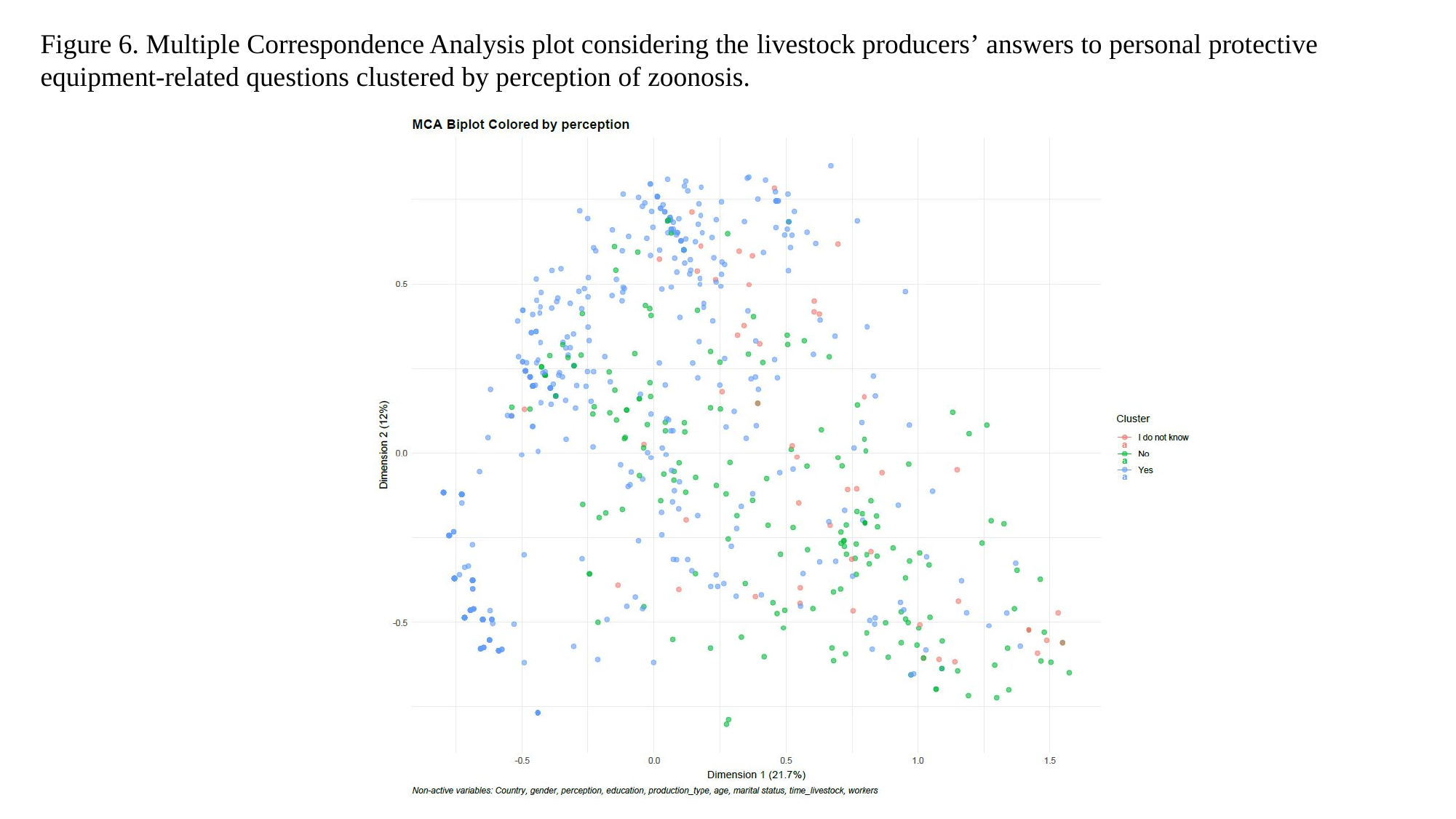

Figure 6. Multiple Correspondence Analysis plot considering the livestock producers’ answers to personal protective equipment-related questions clustered by perception of zoonosis.

## Slide 7
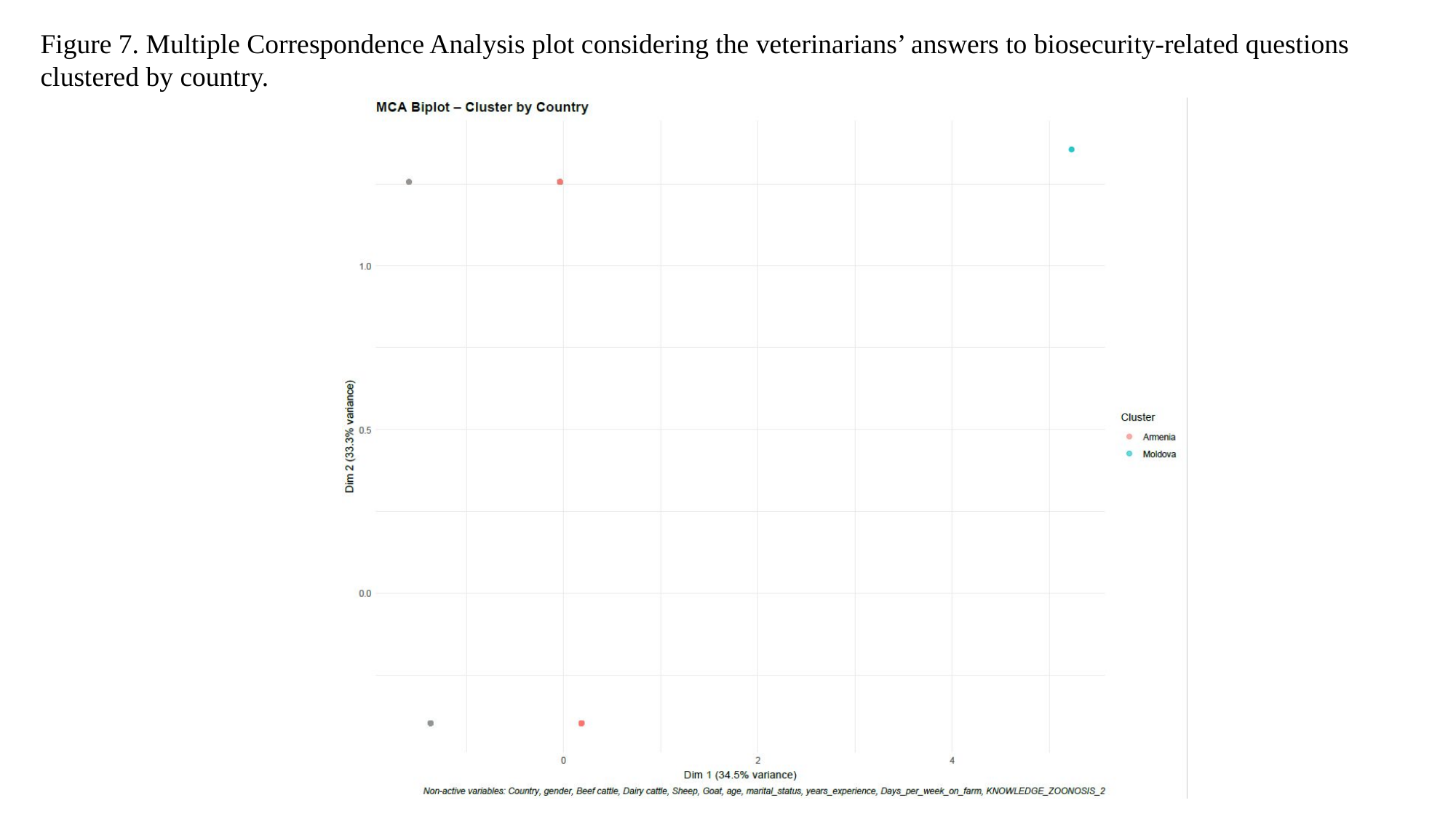

Figure 7. Multiple Correspondence Analysis plot considering the veterinarians’ answers to biosecurity-related questions clustered by country.

## Slide 8
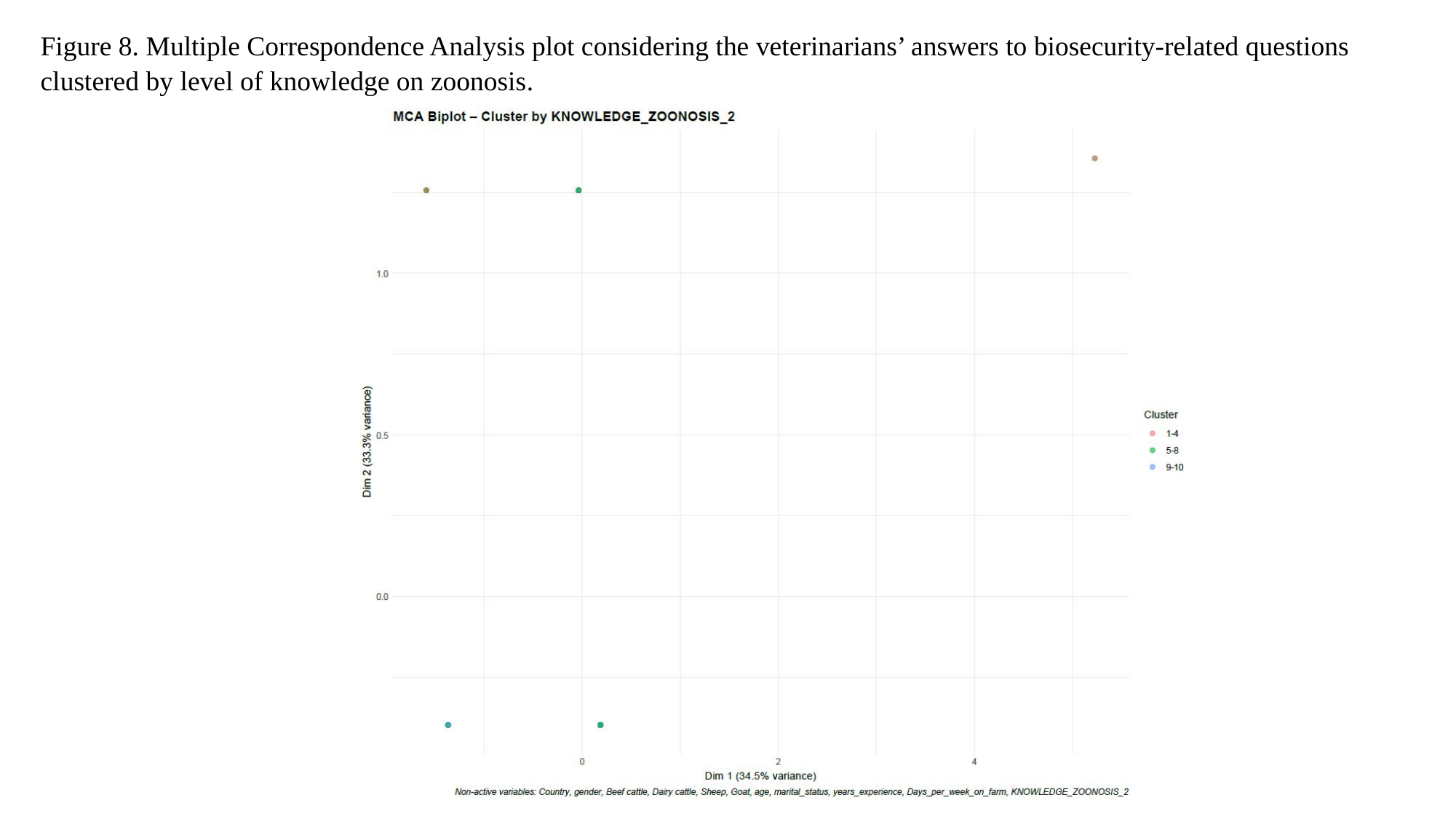

Figure 8. Multiple Correspondence Analysis plot considering the veterinarians’ answers to biosecurity-related questions clustered by level of knowledge on zoonosis.

## Slide 9
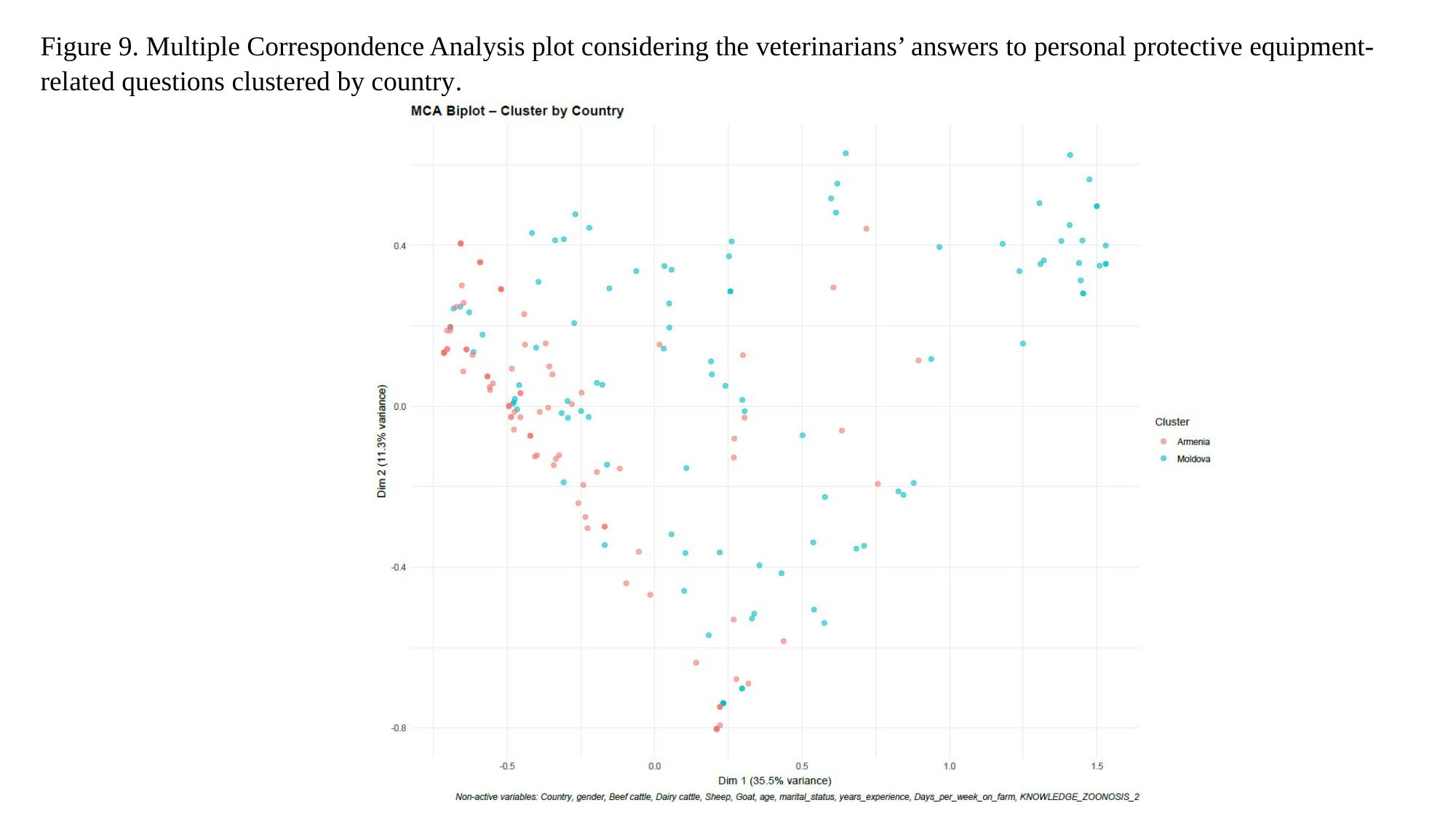

Figure 9. Multiple Correspondence Analysis plot considering the veterinarians’ answers to personal protective equipment-related questions clustered by country.

## Slide 10
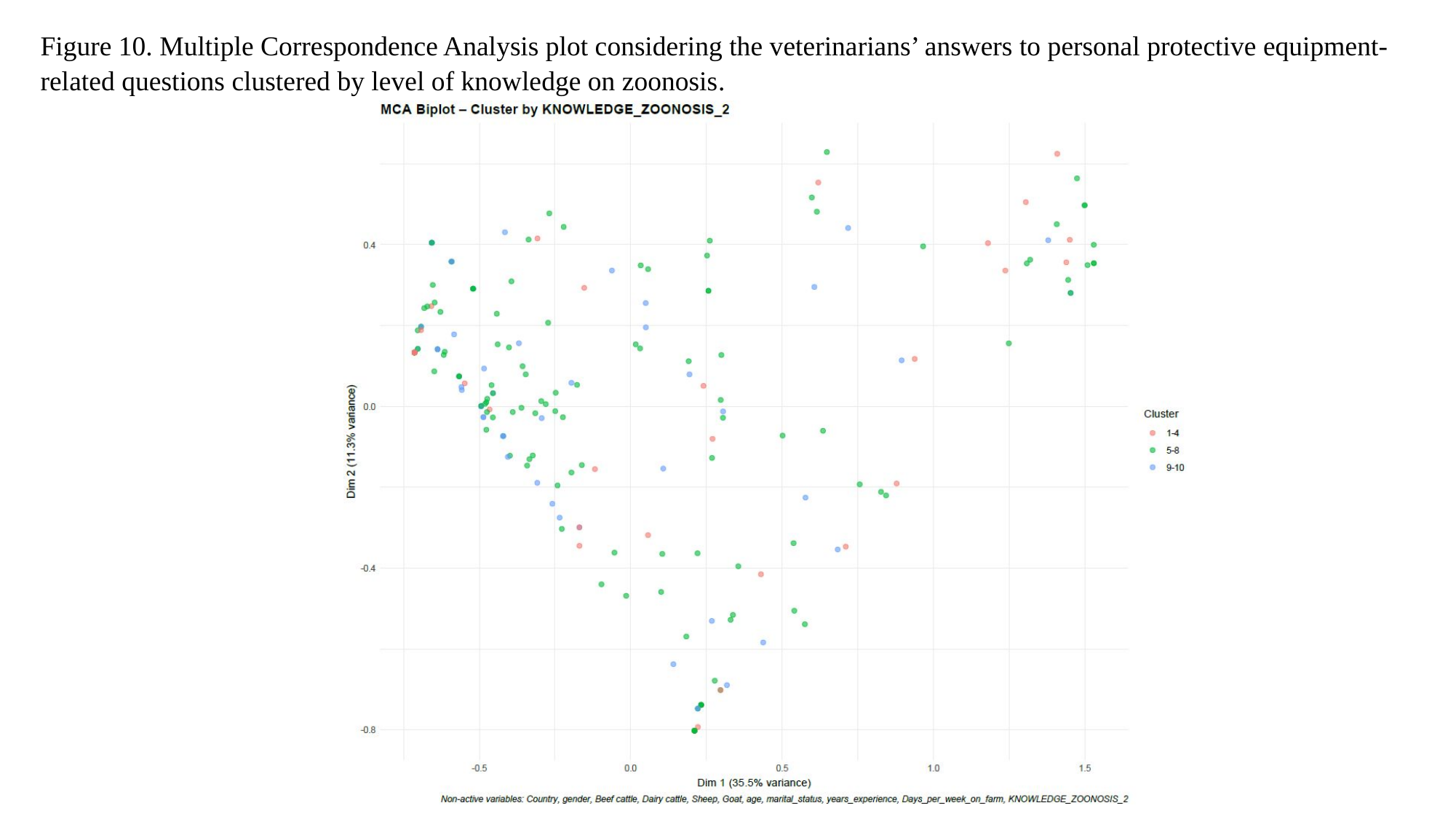

Figure 10. Multiple Correspondence Analysis plot considering the veterinarians’ answers to personal protective equipment-related questions clustered by level of knowledge on zoonosis.
